# Genome-wide analysis of R2R3-MYB transcription factors in poplar and functional validation of PagMYB147 in defense against *Melampsora magnusiana*

**DOI:** 10.1007/s00425-024-04458-3

**Published:** 2024-07-06

**Authors:** Bin Wang, Chaowei Xiong, Zijia Peng, Zeyu Luo, Xiujuan Wang, Shaobing Peng, Zhongdong Yu

**Affiliations:** 1 College of Forestry, Northwest A & F University, Yangling, 712100 Shaanxi China; 2 College of Plant Protection, Gansu Agricultural University, Lanzhou, 730070 Gansu China

**Keywords:** Biotic stress, Disease resistance, Leaf rust disease, Phytohormones, Plant–pathogen interaction, *P. alba* × *P. glandulosa*, R2R3-MYBs, ROS homeostasis

## Abstract

**Main conclusion:**

Transcription of *PagMYB147* was induced in poplar infected by *Melampsora magnusiana*, and a decline in its expression levels increases the host's susceptibility, whereas its overexpression promotes resistance to rust disease.

**Abstract:**

Poplars are valuable tree species with diverse industrial and silvicultural applications. The R2R3-MYB subfamily of transcription factors plays a crucial role in response to biotic stresses. However, the functional studies on poplar R2R3-MYB genes in resistance to leaf rust disease are still insufficient. We identified 191 putative R2R3-MYB genes in the *Populus trichocarpa* genome. A phylogenetic analysis grouped poplar R2R3-MYBs and *Arabidopsis* R2R3-MYBs into 33 subgroups. We detected 12 tandem duplication events and 148 segmental duplication events, with the latter likely being the main contributor to the expansion of poplar R2R3-MYB genes. The promoter regions of these genes contained numerous *cis*-acting regulatory elements associated with response to stress and phytohormones. Analyses of RNA-Seq data identified a multiple R2R3-MYB genes response to *Melampsora magnusiana* (*Mmag*). Among them, *PagMYB147* was significantly up-regulated under *Mmag* inoculation, salicylic acid (SA) and methyl jasmonate (MeJA) treatment, and its encoded product was primarily localized to the cell nucleus. Silencing of *PagMYB147* exacerbated the severity of *Mmag* infection, likely because of decreased reactive oxygen species (ROS) production and phenylalanine ammonia-lyase (PAL) enzyme activity, and up-regulation of genes related to ROS scavenging and down-regulation of genes related to PAL, SA and JA signaling pathway. In contrast, plants overexpressing *PagMYB147* showed the opposite ROS accumulation, PAL enzyme activity, SA and JA-related gene expressions, and improved *Mmag* resistance. Our findings suggest that PagMYB147 acts as a positive regulatory factor, affecting resistance in poplar to *Mmag* by its involvement in the regulation of ROS homeostasis, SA and JA signaling pathway.

**Supplementary Information:**

The online version contains supplementary material available at 10.1007/s00425-024-04458-3.

## Introduction

The MYB family of transcription factors (TFs) is a widely distributed and functionally important group of TFs found in eukaryotes. Members of this family are characterized by the presence of a highly conserved MYB domain, which plays a crucial role in DNA binding and the regulation of gene expression. The MYB domain typically consists of one to four incompletely repeating amino acid sequence repeats (R), each containing 52 amino acids and forming three alpha helices. Each R sequence, along with the helix-turn-helix motif, contains conserved amino acid sequences that enable the MYB TFs to bind to specific DNA sequences with high specificity. In plants, the MYB family can be classified into four subfamilies based on the number of MYB repeats: R1-MYB, R2R3-MYB, R1R2R3-MYB, and R1R2R2R1/2-MYB (Dubos et al. 2010).

R2R3-MYB is the largest subfamily within the MYB family, and its members significantly affect plant growth, development, and secondary metabolism (Chopy et al. 2023; Matías-Hernández et al. 2017; Wang et al. 2019a, b, c). R2R3-MYB TFs also play a significant role in plant disease resistance. For instance, tomato SlMYB49 inhibits the production of reactive oxygen species (ROS), thereby enhancing resistance against *Phytophthora infestans* (Cui et al. 2018). *Arabidopsis* AtMYB96 enhances resistance to *Pseudomonas syringae* infection by regulating genes associated with the salicylic acid (SA) pathway (Seo and Park 2010). Wheat TaMYB391 and TaMYB29 strengthen resistance to *Puccinia striiformis* f. sp. *tritici* by modulating the balance of ROS and the SA pathway (Zhu et al. 2021; Hawku et al. 2022). In both, *Arabidopsis* and cotton, MYB36 promotes the activation of the *PR1* gene in the SA pathway, thereby enhancing resistance against *Verticillium dahliae* (Liu et al. 2021). Together, the results of those studies show that R2R3-MYB TFs, through their involvement in regulating plant-pathogen interactions and activating defense responses, enhance plants’ resistance to pathogens.

Poplar is a widely distributed deciduous tree with multiple ecological and economic values and was chosen as a model species for studying woody plants for its simple genetic characteristics, robust reproductive capacity, and small genome size (Eberl et al. 2018). However, poplar suffers from heavy rust disease caused by the obligate biotrophic pathogen *Melampsora*, which may result in premature leaf abscission in young seedlings, and up to 50% reduction in biomass (Polle et al. 2013; Wan et al. 2013). R2R3-MYB TFs regulate the response of poplar to pathogenic fungi. For example, MYB115 increases resistance to *Dothiorella gregaria* by regulating the biosynthesis of proanthocyanidins (Wang et al. 2017). Overexpression of *PalMYB90* and *PalbHLH1* in poplar enhances resistance to *Botrytis cinerea* and *Dothiorella gregaria* by regulating the flavonoid pathway and ROS pathway (Bai et al. 2020). Overexpression of *MYB134* enhances proanthocyanidins accumulation, thereby increasing resistance to *Melampsora larici-populina* infection in poplar (Ullah et al. 2017). Additionally, RNA-seq analysis identified many R2R3-MYB genes responsive to *M. larici-populina* in poplar, but their functions have not been validated (Chen et al. 2022). Although a total of 192, 196, or 207 R2R3-MYB TFs have been identified across various genome versions, it is challenging to locate the IDs for some of these genes in the latest database version (Wilkins et al. 2008; Zhao et al. 2020; Yang et al. 2021). To date, there haven't been reports on the identification and characterization of the R2R3-MYB family in the latest version of the poplar genome and its response to rust fungus.

In this study, we performed a comprehensive analysis of the R2R3-MYB TF subfamily in poplar. The molecular features, chromosomal localization, phylogenetic relationships, gene structure, conserved motifs, synteny, *cis*-acting elements and transcriptional profiles of *PtrMYB* genes were determined. We identified a significantly up-regulated gene during *M. magnusiana* (*Mmag*) inoculation, *PagMYB147*, and determined its transcription patterns in response to *Mmag*, salicylic acid (SA), and methyl jasmonate (MeJA). To gain deeper insights into the role of PagMYB147 in rust resistance, we conducted experiments using virus-induced gene silencing (VIGS) and overexpressed methods in poplar. The changes in certain physiologic and biochemical traits, as well as the transcript levels of defense-related genes, were analyzed after *Mmag* inoculation. These findings provide valuable insights into the roles of poplar R2R3-MYB TFs in the response to biotic stress.

## Materials and methods

### Identification of R2R3-MYB TFs in *P. trichocarpa*

The *P. trichocarpa* genome sequencing project was accessed from the Phytozome database (https://phytozome-next.jgi.doe.gov/). The MYB protein sequence from *A. thaliana* was obtained from the TAIR database (http://www.arabidopsis.org). We used two methods to maximize the identification of R2R3-MYBs. The first method involved using *A. thaliana* R2R3-MYBs protein sequences as search queries in a BLASTp search to identify R2R3-MYBs in the *P. trichocarpa* genome. The second method leveraged a hidden Markov model of MYB DNA-binding domain (PF00249) downloaded from the Pfam database (http://pfam.xfam.org/) to identify MYB candidate genes in the *P. trichocarpa* genome (El-Gebali et al. 2018). Subsequently, SMART (http://smart.embl-heidelberg.de/) was used to scrutinize the conserved structures of all potential proteins with a threshold *e* value < 0.0001 to identify all the MYB candidate genes in the *P. trichocarpa* genome. The location of each gene was determined by TBtools, and then genes were named according to their position on the *P. trichocarpa* chromosomes (Chen et al. 2020). The characteristics of the putative proteins, including their isoelectric point, molecular weight, grand average hydrolysis (GRAVY), and instability index were predicted using ProtParam (https://web.expasy.org/protparam/) and TBtools (Chen et al. 2020). Signal peptides and phosphorylation sites were predicted using tools at the SignalP 5.0 server and NetPhos 3.1 server (http://www.cbs.dtu.dk/services/NetPhos/), respectively. Plant-mPLoc (http://www.csbio.sjtu.edu.cn/bioinf/plant-multi/) was used to predict the subcellular localization of the identified gene products.

### Phylogenetic and synteny analyses of R2R3-MYBs in *P. trichocarpa*

The genomic coordinates of *P. trichocarpa* R2R3-MYBs were retrieved from the genome database, and a visual representation of the data was created using TBtools (Chen et al. 2020). Using the neighbor-joining (NJ) method of MEGA7 and 1000 bootstrap values, a phylogenetic tree was constructed from the protein sequences of R2R3-MYBs in *P. trichocarpa* and *A. thaliana*. The generated tree was visualized using iTOL (https://itol.embl.de/) (Letunic and Bork 2019). Gene duplication events were detected using TBtools, BLASTP, and the Multiple Collinearity Scan (MCScan) toolkit (Zhao et al. 2020). Tandem duplication events were indicated by the presence of two genes located within the same chromosomal segment and not separated by more than five genes within a 100-kb region (Li et al. 2022). Finally, a synteny map of R2R3-MYBs in *P. trichocarpa*, *A. thaliana* (dicots), and *Oryza sativa* (a monocot), was generated using the Dual Synteny Plotter software (Chen et al. 2020).

### Analysis of R2R3-MYBs conserved motifs, gene structure, and *cis*-acting elements upstream of R2R3-MYB genes in *P. trichocarpa*

The online software MEME (https://meme-suite.org/meme/tools/meme) was used to find conserved motifs in *P. trichocarpa* R2R3-MYBs (Bailey et al. 2009). TBtools was used to analyze and show intron/exon structures (Chen et al. 2020). The PlantCARE database (http://bioinformatics.psb.ugent.be/webtools/plantcare/html/) was used to identify *cis*-acting elements associated with stress and phytohormone responsiveness in the promoter region (2 kb upstream) of each R2R3-MYB gene. The distribution of these elements was visualized with TBtools (Chen et al. 2020).

### Plant materials and treatments

One-month-old sterile seedlings of the 84 K poplar (*P.alba* × *P.glandulosa*) were transplanted into commercial substance soil and cultivated in a greenhouse. For one month, the conditions were strictly controlled at 25 °C, 50%-60% relative humidity, and a 16-h light/8-h dark photoperiod. Uniformly sized seedlings were selected, and the leaves were washed softly with sterile water before inoculation. A suspension of fresh *Mmag* uredospore (1 × 10^5^ spores/mL), or water as the control, was sprayed on abaxial leaves with a leaf plastochron index (LPI) of 5–10 (Ullah et al. 2018). The plants were then placed in a light-proof container and kept in the dark for 24 h, and then returned to the same growth conditions as described above. Leaf samples were collected and immediately frozen in liquid nitrogen at 0, 24, and 144 h post-inoculation (hpi). The experiment was designed with three independent biologic replicates, and at least three leaf samples were collected at each time point. Each sample was subjected to RNA-Seq sequencing to pick up the putative MYB related to rust resistance. All rust infestation experiments were conducted using the same inoculation and sampling procedures unless otherwise noted.

### Expression analysis of poplar R2R3-MYB genes

To analyze the transcriptional profiles of R2R3-MYB genes in 84 K poplar, transcriptome data (unpublished data) obtained from plants after inoculation with *Mmag* were obtained, and gene transcript levels were calculated using fragments per kilobase of transcript per million fragments mapped (FPKM) values. A gene clustering heatmap was generated using the TBtools HeatMap tool. In addition, differentially expressed genes (DEGs) were identified by selecting those with a Log_2_|foldchange|≥ 1, and these DEGs under infection conditions were compared to control samples (Chen et al. 2020). For validation, fourteen genes were selected for quantitative real-time polymerase chain reaction (qRT-PCR) analysis. The transcript levels of these genes were determined by qRT-PCR using 2 × SYBR Green qPCR Master Mix (US Everbright, Suzhou, China) on a CFX-96 system (Bio-Rad, Hercules, CA, USA). Total RNA was extracted using the E.Z.N.A.® Plant RNA Kit (OMEGA, Norcross, GA, USA), and cDNA was synthesized using UEIris RT mix with DNase (US Everbright). The relative gene expression levels were determined using the 2^−ΔΔCt^ method and normalized to that of the endogenous reference *actin* gene (Livak and Schmittgen 2001). The primer sequences, which were designed with Primerquest (https://sg.idtdna.com/pages/tools/primerquest), are listed in Table S1.

### Cloning and characterization of *PagMYB147* in 84 K poplar

We selected *MYB147* and designed primers based on the *PtrMYB147* (Potri.015G046200) sequence. Initially, RNA was extracted from the leaves of 84 K poplar plants, cDNA was synthesized, and PCR amplification was subsequently performed. The product was purified, ligated into the pMD-19 T vector and sequenced. The subcellular localization was analyzed by inserting the open reading frame sequence of the *PagMYB147* gene, without the termination codon, into the EGFP vector (Miaoling Biology, Wuhan, China) under the control of the CaMV35S promoter. The resulting fusion plasmid was then transformed into competent cells of *Agrobacterium tumefaciens* GV3101 (Weidi Biotechnology, Shanghai, China), which were then used to introduce the gene construct into one-month-old *Nicotiana benthamiana* seedlings for transient expression. The inner epidermis of tobacco specimens was injected with *A. tumefaciens* buffer containing 35S-EGFP and 35S-*PagMYB147*-EGFP. The plants were then incubated in darkness for 24 h. After 48 h, a confocal laser scanning microscope (Olympus, Tokyo, Japan) was used to observe the fluorescence of the heterologously expressed protein in the leaves. The primer sequences used for gene cloning and vector construction are described in Table S1. To determine the transcriptional profile of *PagMYB147*, qRT-PCR was conducted on untreated roots, stems, young and mature leaves, as well as leaves of 84 K poplar plants that had been treated with *Mmag*, SA (5 mM) and MeJA (1 mM).

### Construction of virus-induced gene silencing (VIGS) and overexpression vectors and genetic transformation of poplar

The *PagMYB147* gene was amplified using specific primers by PCR, and then integrated into the pTRV2 vector. Then, pTRV2-*PagMYB147* was introduced into *A. tumefaciens* GV3101, and the pTRV2 empty vector was introduced into *A. tumefaciens* as the control. The VIGS experiments were conducted in accordance with the protocol outlined by Shen et al. (2015). The transcript levels of *PagMYB147* were detected by qRT-PCR in transformed with *A. tumefaciens* harboring pTRV2-*PagMYB147* and pTRV2. Silenced plants displaying significantly reduced *PagMYB147* levels were selected for disease resistance testing.

The *PagMYB147* sequence was cloned into the pCAMBIA-1300 vector. The recombinant plasmid was then transformed into *A. tumefaciens* GV3101. Subsequently, the transformed *A. tumefaciens* was used for leaf disk transformation into the 84 K poplar (He et al. 2018). Initially, transgenic lines were selected based on their resistance to hygromycin, and then the presence of the transgene was confirmed by PCR confirmation using specific primers (Table S1). The relative transcript level of *PagMYB147* in the transgenic lines was further examined using qRT-PCR. One-month-old transgenic seedlings of the 84 K poplar were transplanted according to the method described in the 'plant material and treatment' section, and were subsequently subjected to rust resistance testing.

### Analysis of disease resistance, physiological parameters, and defense gene expression

To validate the disease resistance function of *PagMYB147*, *PagMYB147*-silenced plants, *PagMYB147*-overexpressing (OE) plants and wild-type (WT) plants were inoculated with *Mmag*. Three independent biologic replicates were prepared and analyzed for each treatment. Leaves were collected at 0, 24, and 48 hpi, and then immediately frozen in liquid nitrogen, and subsequently, stored in an ultra-low temperature freezer until further analysis. Phenotypic observations and evaluation of uredinial density (number of uredinia/cm^2^) were conducted 10 d post-inoculation (dpi). Using qRT-PCR to determine the transcription level of the *Mmag ITS* gene to determine the biomass of the rust fungus, with *ELF1α* as reference genes (Table S1) (Zheng et al. 2021). The contents of H_2_O_2_, along with the activities of superoxide dismutase (SOD, EC:1.15.1.1), catalase (CAT, EC 1.11.1.6), and phenylalanine ammonia-lyase (PAL, EC 4.3.1.5) were determined using previously described methods (Li et al. 2015b). The relative transcript levels of *PtrSOD1*, *PtrCAT1*, *PtrPAL1*, *PtrPR5*, and *PtrDefensin* were also determined by qRT-PCR using the primers specified in Table S1. The PlantCARE program was used to analyze the MYB, MYB binding sites (MBS) and MYB recognition elements (MRE) within the upstream 2 kb region of these genes, which were considered to contain complete promoters (Lescot 2002).

### Statistical analysis

All data were shown as means with standard deviations (SD), calculated from three independent biologic replicates. Statistical analyses were conducted using SPSS version 26.0 software (IBM Corp., Chicago, Ill., USA). To detect significant differences among groups, a one-way analysis of variance (ANOVA) was conducted, followed by Duncan’s multiple range test, with a significance threshold value of *P* < 0.05.

## Results

### Identification of R2R3-MYB genes in the *P. trichocarpa* genome and physical properties of their putative products

**Fig. 1:**
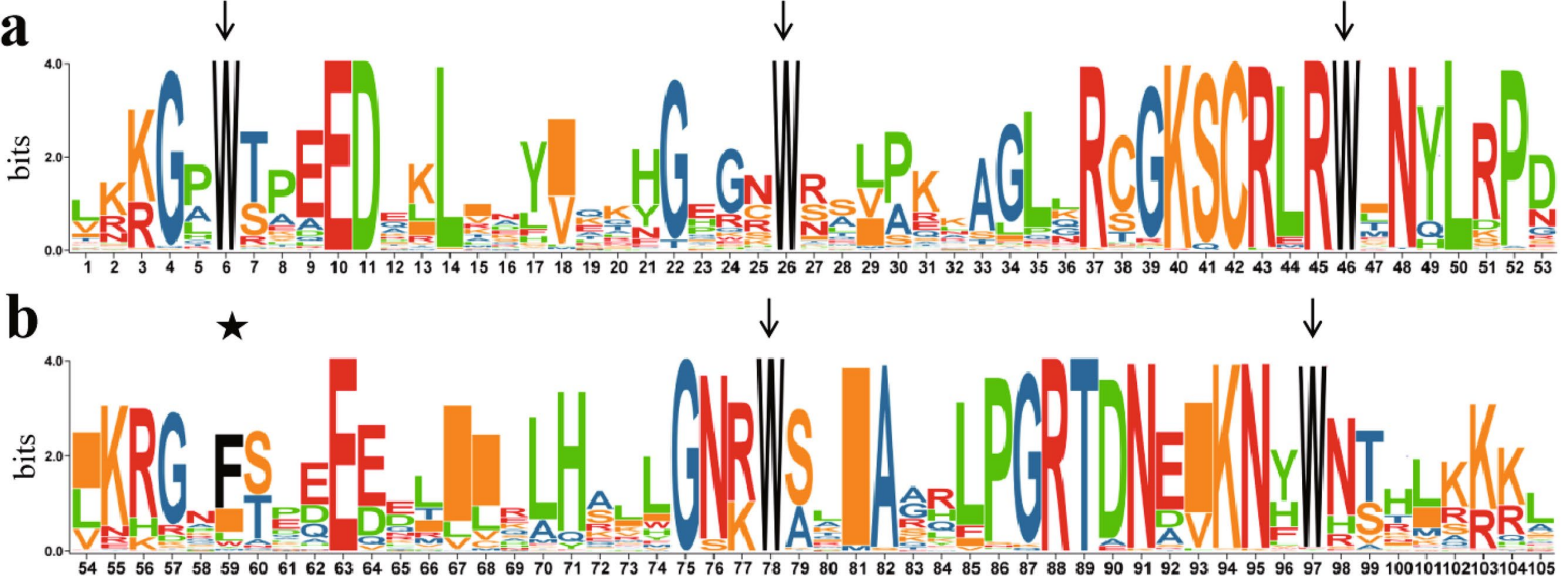
The sequence logos of *P. trichocarpa* R2 **a** and R3 **b** MYB domains. The arrow indicates typically conserved tryptophan (Trp, W) and the star indicates the first W residues can be replaced by phenylalanine (Phe, F), isoleucine (Ile, I) and leucine (Leu, L)

In total, 191 R2R3-MYB genes were identified in the *P. trichocarpa* genome. These genes were named *PtrMYB001* to *PtrMYB190.1* based on their chromosomal locations. Comparison of the amino acid sequences of the R2 and R3 MYB repeats revealed three conserved tryptophan (Trp, W) residues in the R2 repeat. The second and third W residues were conserved in the R3 repeat (Fig. 1a), whereas the first could be replaced by phenylalanine (Phe, F), isoleucine (Ile, I), or leucine (Leu, L) (Fig. 1b). The lengths and physical traits of the putative *P. trichocarpa* R2R3-MYBs are summarized in Table S2. The R2R3-MYBs had an average length of 321 amino acids, ranging from 180 (PtrMYB035) to 568 (PtrMYB042). The molecular weights of the putative proteins ranged from 20.52 kDa (PtrMYB035) to 62.68 kDa (PtrMYB002), and the isoelectric points ranged from 4.72 (PtrMYB136) to 9.74 (PtrMYB079). Of the 191 R2R3-MYBs, 104 had an isoelectric point below 7.0 and 87 had an isoelectric point above 7.0. All R2R3-MYBs were hydrophilic, according to predicted GRAVY scores, which ranged from − 1.067 (PtrMYB119) to − 0.282 (PtrMYB175). The putative R2R3-MYB proteins had multiple phosphorylation sites (Fig. S1), were primarily located in the nucleus, and lacked signal peptides. Of the 191 putative R2R3-MYBs, 186 were categorized as unstable (instability index > 40). The instability index of the putative R2R3-MYBs ranged from 34.25 (PtrMYB007) to 72.65 (PtrMYB147).

### Phylogenetic connections, chromosomal locations, and synteny assessments of* P. trichocarpa* R2R3-MYBs

**Fig. 2:**
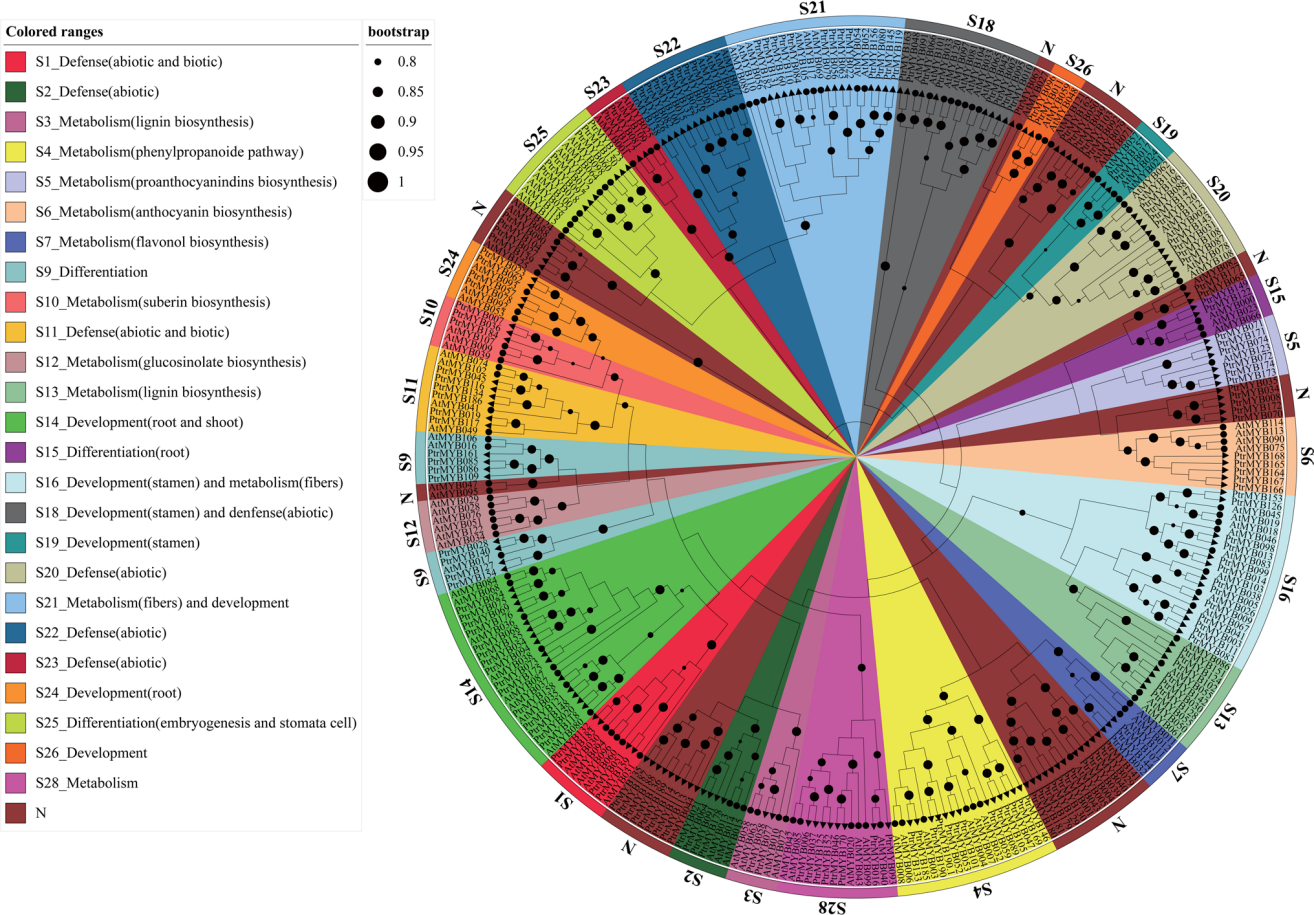
Phylogenetic relationship between* A. thaliana* and *P. trichocarpa* R2R3 MYB TFs. Subgroups are marked with a different background color. This annotation comes from genetic function in *A. thaliana*

We performed a phylogenetic analysis to explore the relationships between the R2R3-MYBs of two plant species, *A. thaliana* and *P. trichocarpa*. The categorization system used for *A. thaliana* R2R3-MYBs resulted in 33 subgroups in the phylogenetic tree. Notably, *A. thaliana* R2R3-MYB members made up the whole of Subgroup 12 (S12) (Fig. 2; Table S3). We grouped *P. trichocarpa* R2R3-MYBs in accordance with *A. thaliana* R2R3-MYBs with known functions to investigate their potential roles (Fig. 2). This grouping suggested that S1, S2, S11, S20, S22, and S23 were mostly engaged in defense responses while S3, S4, S5, S6, S7, S10, S12, S13, and S28 had a primary roles in regulating metabolism, herein MYB147 was involved in S20. Additionally, S9, S15, and S25 were linked to tissue differentiation or plant development, as were S14, S16, S18, S19, S21, S24, and S26 (Table S3).

**Fig. 3:**
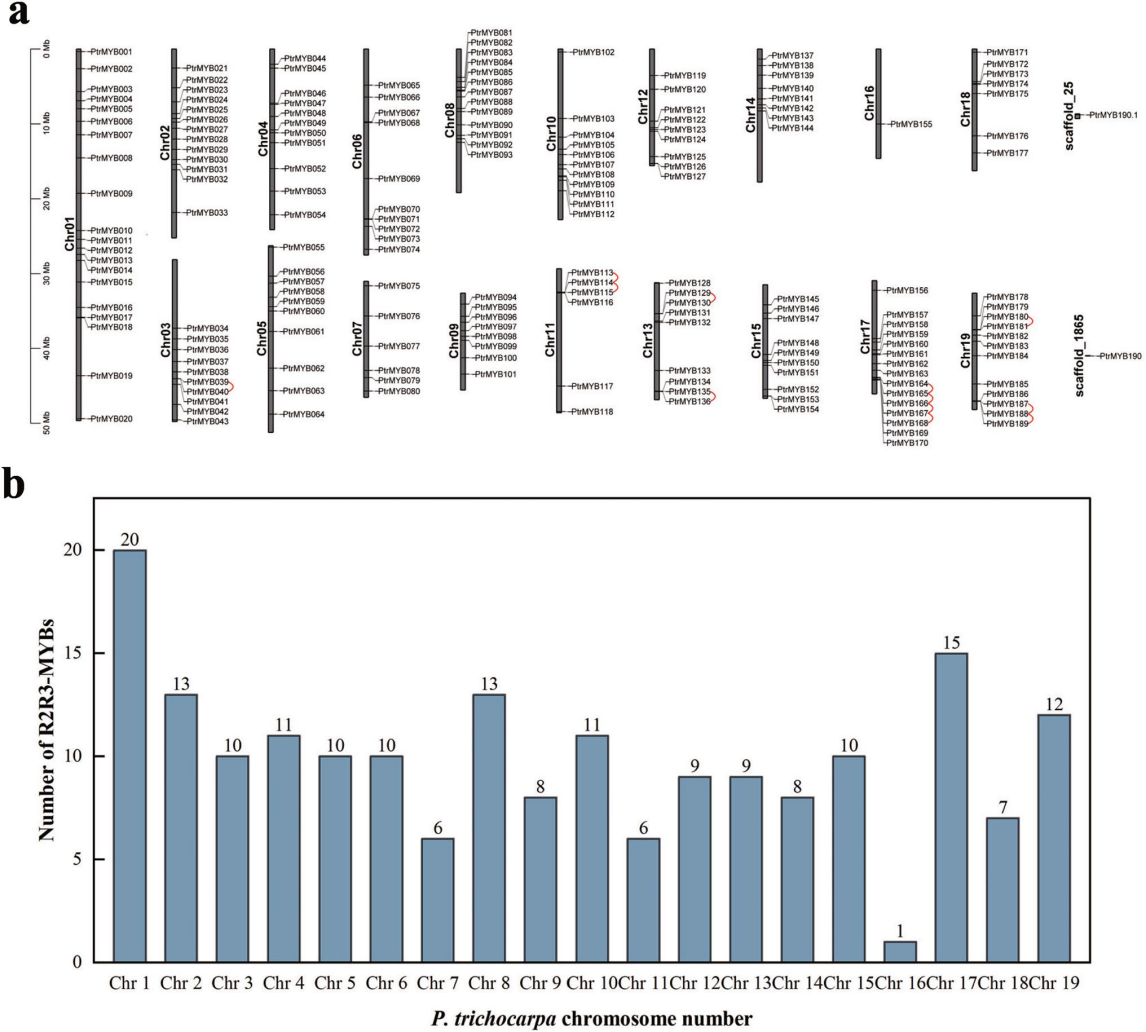
Chromosomal locations. **a** Chromosome distribution of *P. trichocarpa* R2R3-MYB genes. **b** Numbers of *P. trichocarpa* R2R3-MYB genes on each chromosome

**Fig. 4:**
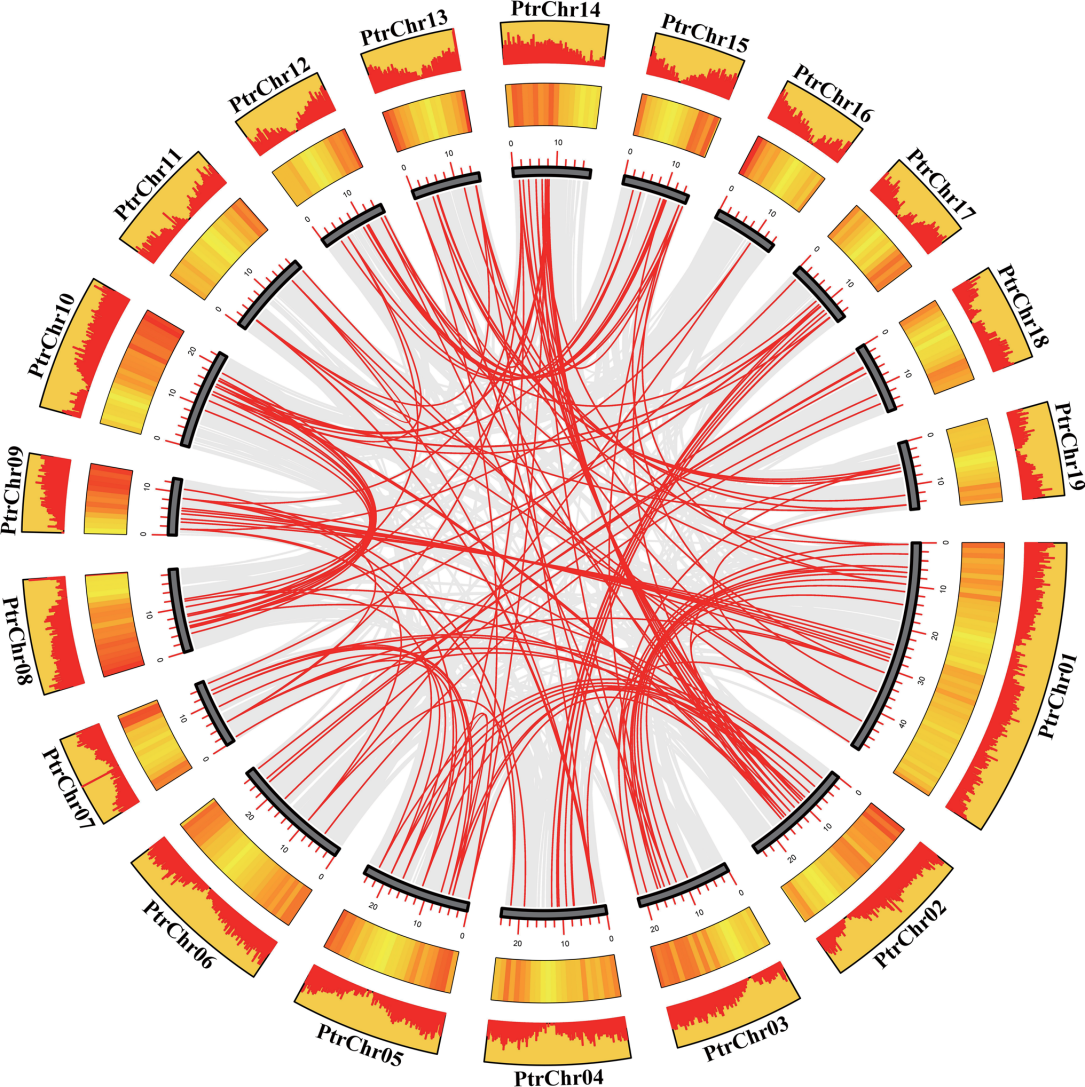
Duplication events between R2R3-MYB members in *P. trichocarpa*. Red lines between chromosomes and gray lines in the background indicate segmental duplicate gene pairs and all synteny blocks in the *P. trichocarpa* genome, respectively. Yellow areas represent the gene density on chromosomes

Next, we investigated the chromosomal distribution of R2R3-MYB genes in *P. trichocarpa* (Fig. 3a). Of the 191 R2R3-MYB genes, 189 were widely dispersed among the 19 chromosomes of *P. trichocarpa*, and two were located on the scaffold. Notably, chromosome 1 had the highest number of genes, with 20 R2R3-MYB genes, while chromosome 16 had the lowest number, with only one gene. The number of genes on other chromosomes ranged from six to 15 (Fig. 3b). In total, we identified 160 pairs of gene duplicates across 15 chromosomes, consisting of tandem duplicates and segmental duplicates or whole-genome duplication (WGD) duplicates. The distribution of these duplicates was uneven (Table S4). Twelve tandem duplication events involving 19 *P. trichocarpa* R2R3-MYBs genes were identified on chromosomes 3, 11, 13, 17, and 19. Although most of the tandem repeats comprised duplicated or triplicate genes, one notable tandem repeat on chromosome 17 harbored five genes (Fig. 3a). Moreover, 148 segmental duplication events were observed across 19 chromosomes, involving 158 R2R3-MYB genes (Fig. 4; Table S4). These results firmly establish the possibility that segmental duplication has had an important effect on the evolutionary process of the R2R3-MYB family in *P. trichocarpa*.

**Fig. 5:**
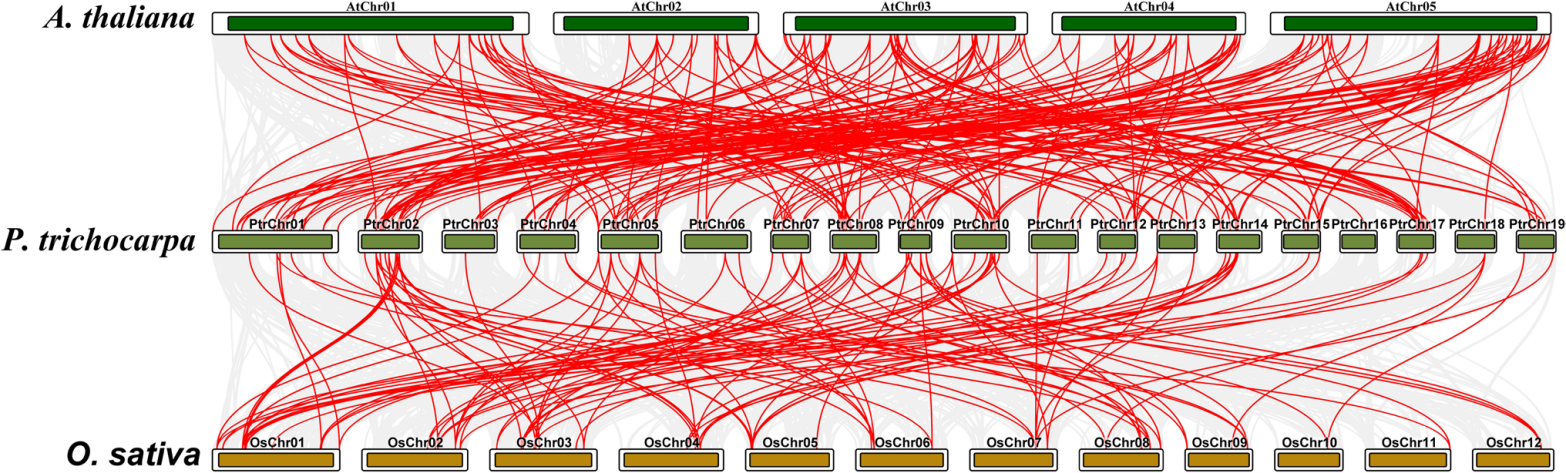
Synteny analysis of R2R3-MYB genes between *P. trichocarpa* and two monocotyledonous and dicotyledonous plants (*O. sativa* and *A. thaliana*). Red lines indicate syntenic R2R3-MYB gene pairs in the genomes of *P. trichocarpa* and other model plants. Gray lines indicate syntenic blocks

We examined the synteny among *P. trichocarpa*, *O. sativa*, and *A. thaliana* to understand more about the genetic development of R2R3-MYBs (Fig. 5). We detected syntenic links between 67 *P. trichocarpa* R2R3-MYBs and 48 *O. sativa* R2R3-MYBs (Table S5), as well as between 111 *P. trichocarpa* R2R3-MYBs and 92 *A. thaliana* R2R3-MYBs (Table S6). Interestingly, 51 *P. trichocarpa* R2R3-MYBs showed syntenic links with both *O. sativa* and *A. thaliana* (Fig. 5).

### Motifs and gene structures of R2R3-MYB genes in *P. trichocarpa*

**Fig. 6:**
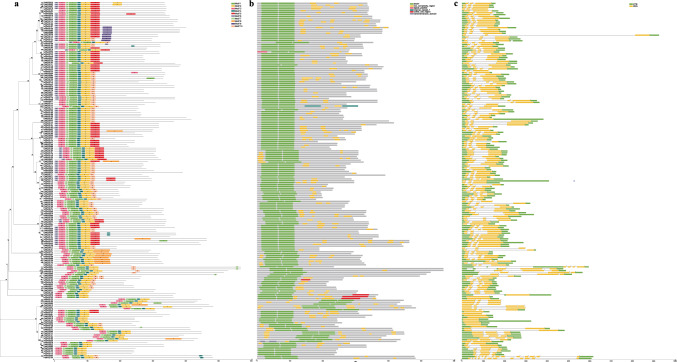
Motif composition, domain structure and gene structure of the R2R3-MYB TFs in *P. trichocarpa*. **a** Evolutionary tree and conserved motifs of R2R3-MYBs in *P. trichocarpa*. Each conserved motif is indicated by a different color block for the first module in the top right. **b** The domain structure of R2R3-MYBs in *P. trichocarpa*. Each domain structure is indicated by a different color block in the top right. **c** Gene structure of R2R3-MYBs in *P. trichocarpa*. Each gene structure is indicated by a different color block at the top right. The coding region, untranslated region, and sant are shown as green boxes, yellow boxes and black lines, respectively

Ten conserved motifs were detected in the 191 *P. trichocarpa* R2R3-MYB genes. These motifs had amino acid lengths ranging from 11 to 50 (Table S7). All of the R2R3-MYBs contained one significantly conserved motif (motif 3), and 188 out of the 191 putative proteins contained two highly conserved motifs (motif 2 and motif 3) (Fig. 6a). These motifs represent critical repetitive components of R2R3-MYBs. These findings demonstrate that all R2R3-MYBs contained the characteristic SANT structural domains that are typical of this gene family (Fig. 6b). A detailed analysis of gene exon/intron structures revealed significant diversity among the 191 genes. Specifically, 11 R2R3-MYB genes (nine distributed between *PtrMYB025* and *PtrMYB138*, as well as *PtrMYB163* and *PtrMYB048*) lacked introns, whereas the remaining 180 genes had varying numbers of introns (ranging from one to 11) (Fig. 6c). These genes within an identical subgroup exhibited a high level of conservation despite differences in the number of introns and exons.

### *Cis*-elements in promoter regions and transcriptional profiles of poplar R2R3-MYB genes

Analysis of the promoter region of *P. trichocarpa* R2R3-MYB genes identified numerous elements involved in responsiveness to various stresses and phytohormones (Fig. S2). These included elements that are known to be involved in responsiveness to stress, defense, phytohormones (abscisic acid (ABA), SA, MeJA), and drought. Of the 191 genes, around 152 had at least one stress-responsive *cis*-element in their promoter regions, which highlighted the importance of the *P. trichocarpa* R2R3-MYB family in biotic stress responses. In addition, 93, 134, and 152 R2R3-MYB genes had *cis*-elements involved in responsiveness to SA, MeJA, and ABA, respectively, in their promoter regions.

**Fig. 7:**
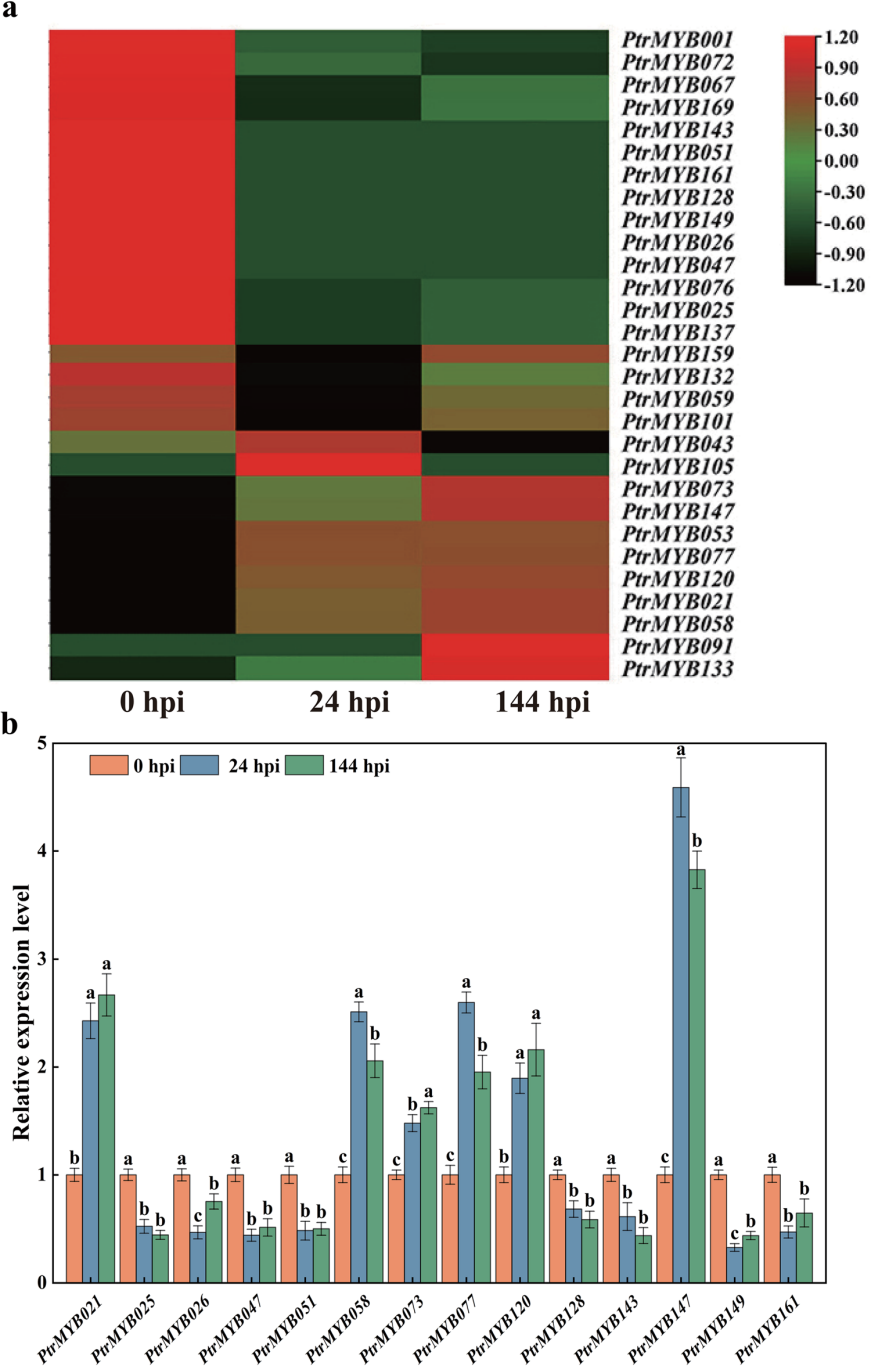
Expression analysis of poplar R2R3-MYB genes in response to *Mmag*. **a** Heatmap and hierarchical clustering of R2R3-MYB gene expression in 84K poplar leaves after inoculation with *Mmag* 0 hpi, 24 hpi, 144 hpi. **b** Expression analysis of fourteen poplar R2R3-MYB genes in response to *Mmag.* The data are expressed as the means ± SD of three biologic replicates. Different lowercase letters indicate significant differences (*P* < 0.05)

To investigate how poplar R2R3-MYB genes respond to rust fungus, we analyzed RNA-Seq data from samples at 24 hpi and 144 hpi with inoculated *Mmag* (Fig. 7a). A heatmap was constructed, revealing 27 DEGs at 24 hpi, with 9 up-regulated and 18 down-regulated genes. Similarly, at 144 hpi, there were 26 DEGs, with 9 up-regulated and 17 down-regulated genes. Comparison of DEGs among different time points identified 24 genes consistently responsive to *Mmag* at both 24 hpi and 144 hpi, consisting of 8 up-regulated and 16 down-regulated genes. To validate RNA-Seq data, we selected 14 genes with fold changes greater than 3 (six up-regulated and eight down-regulated) for qRT-PCR validation. The qRT-PCR results were consistent with those detected from the transcriptome data, validating the reliability of the transcriptome data (Fig. 7b). Of particular interest was *MYB147*, which exhibited a significant increase in its transcript levels in response to *Mmag* at both 24 hpi and 144 hpi. At 24 hpi, *MYB147* expression was up-regulated by 4.59-fold, showing the most substantial increase. Similarly, at 144 hpi, its expression was increased by 3.83-fold. As a result, we selected *MYB147* as the primary candidate for further functional analyses to better understand poplar's response to rust fungus.

### Characterization of PagMYB147

**Fig. 8:**
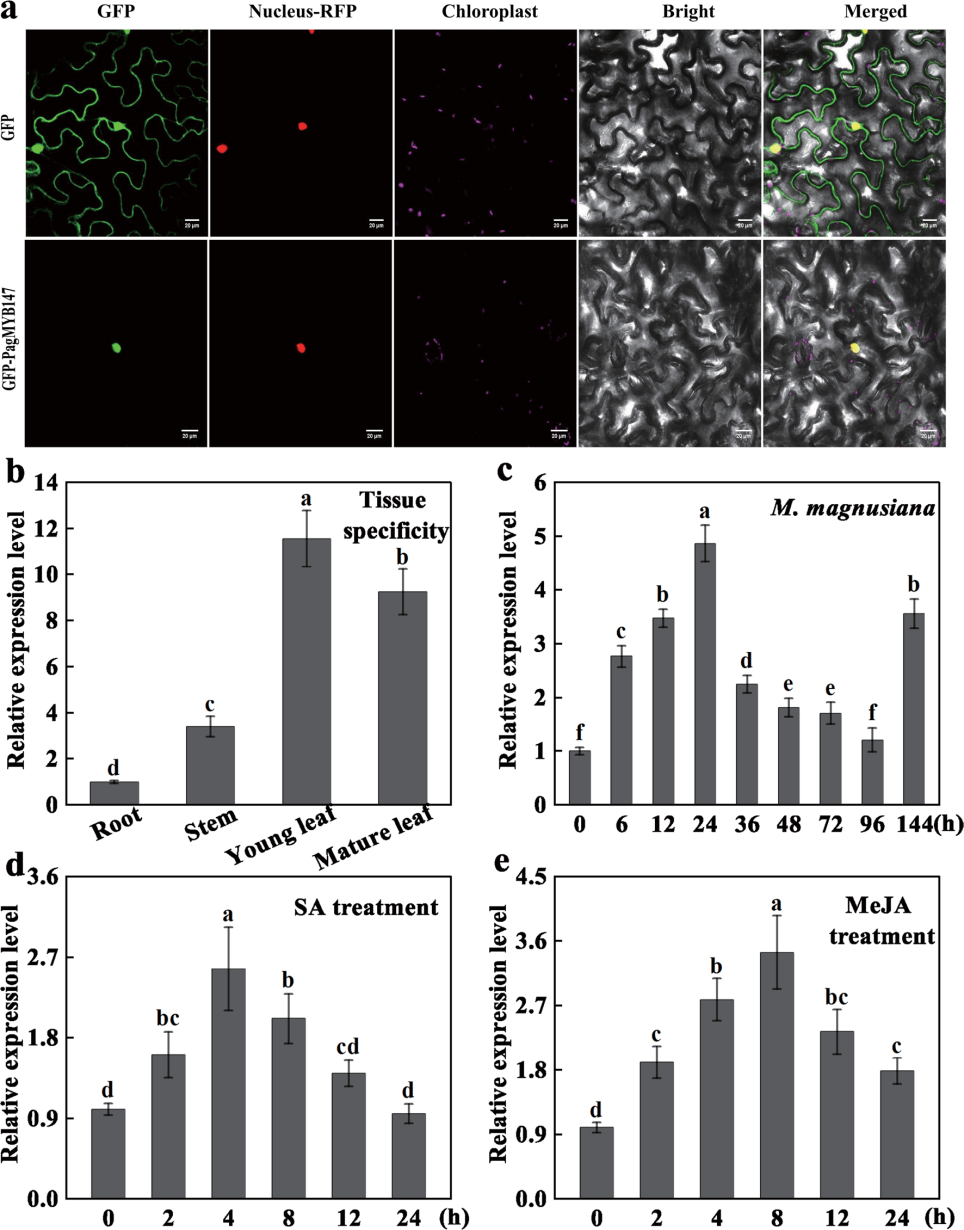
Characterization of PagMYB147. **a** Subcellular localization of PagMYB147. 35S-EGFP was taken as the control and the scale bars were equal to 20 μm. **b** Analysis of tissue-specific expression patterns of *PagMYB147*. **c**-**e** Expression patterns of *PagMYB147* in 84K poplar leaves at the indicated time treated with *Mmag*, plant hormones SA and MeJA, respectively. All data are presented as mean ± SD of three biologic replicates. Different letters above the bars indicate significant differences (*P* < 0.05)

*PagMYB147* had a coding sequence length of 999 bp, encoding 332 amino acids, which was characterized by its instability and hydrophilicity. Through laser confocal microscopy observations, it was determined that the green fluorescence signal of PagMYB147 was primarily concentrated in the nucleus of tobacco leaf epidermal cells (Fig. 8a), indicating that PagMYB147 is localized in the cell nucleus. We then conducted qRT-PCR analyses to investigate the patterns of *PagMYB147* transcription in different 84 K poplar tissues. The transcript levels of *PagMYB147* were higher in the young leaves than in the mature leaves, stems, and roots (Fig. 8b). To investigate the role of *PagMYB147* in poplar defense, we analyzed the expression levels of *PagMYB147* by qRT-PCR within 144 h of inoculation with *Mmag* and 24 h of treatment with SA and MeJA. The findings revealed that the expression of *PagMYB147* was induced by *Mmag*, and its expression levels gradually increased from 0 to 24 hpi. At 24 hpi, the expression of *PagMYB147* reached its peak, being 4.12 times higher than at 0 hpi. Subsequently, the expression levels gradually declined. By 144 hpi, the expression of *PagMYB147* increased once again (Fig. 8c). After treating seedlings with SA (Fig. 8d) and MeJA (Fig. 8e), the transcript levels of *PagMYB147* reached the highest level at 4 h post-treatment (hpt) and 8 hpt, with increases of 2.57 and 3.44 times, respectively. Our findings indicate the involvement of *PagMYB147* in poplar resistance against *Mmag* and diverse hormone signal pathways.

### Rust resistance analyses of *PagMYB147*-silenced and -OE plants

In *PagMYB147*-silenced 84K poplar plants, the transcript level of *PagMYB147* was significantly reduced compared with those in the controls (the empty TRV2 and WT) (Fig. S3). *PagMYB147*-OE plants were detected by PCR analysis using specific primers. As shown in Fig. S4a specific bands were present in the transgenic poplar lines 1–3 and 5–7, while no such positive band was detected in the WT plants. Subsequently, the relative transcript levels of *PagMYB147* in the selected six transgenic lines were detected using qRT-PCR. The three transgenic lines with the highest expression levels (OE 1, OE 5, and OE 7) were used in subsequent experiments (Fig. S4b).

**Fig. 9:**
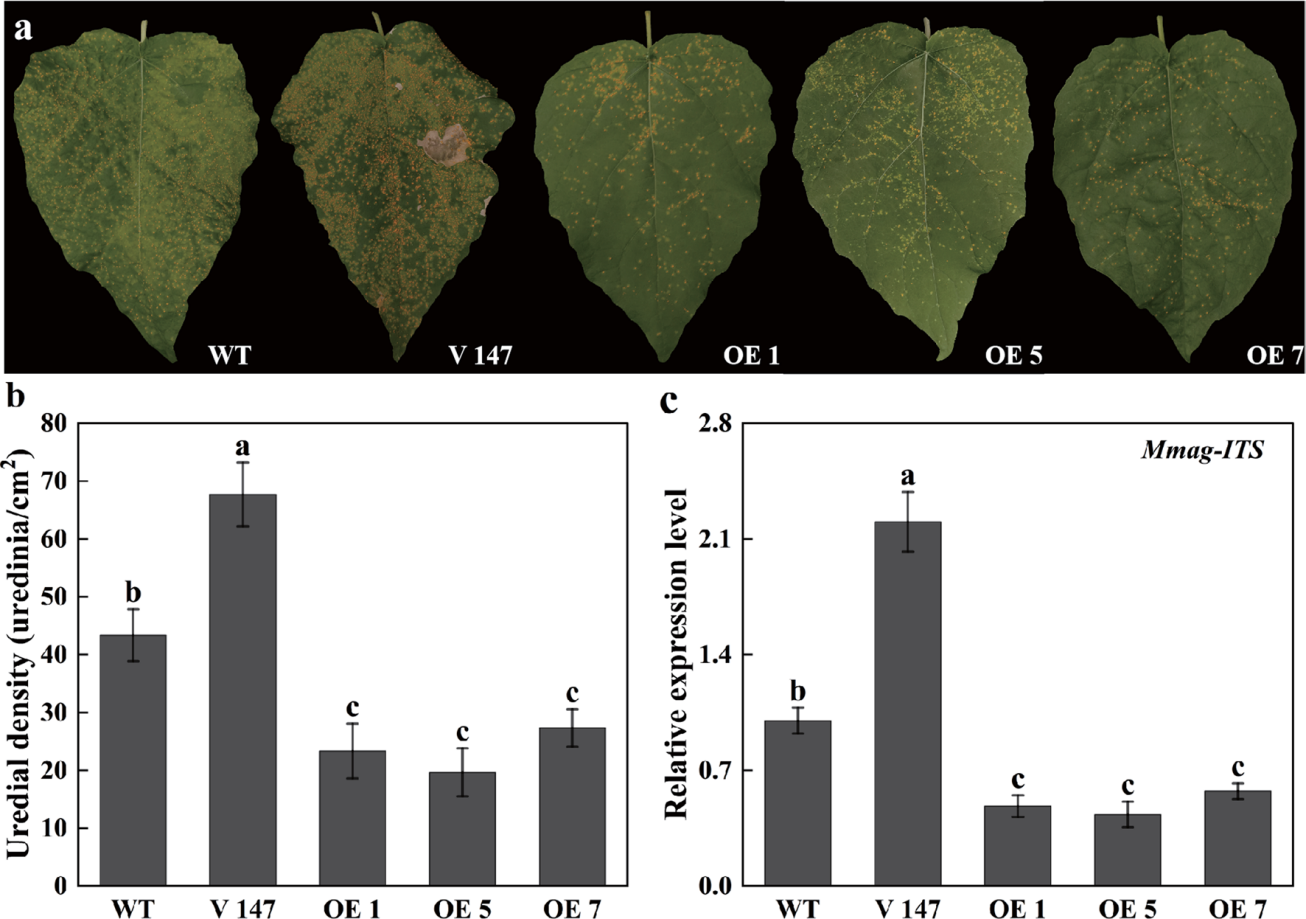
Phenotypes of *PagMYB147* -silenced and -overexpressing plants after inoculation with *Mmag.* **a** Phenotypes of *PagMYB147* -silenced and -overexpressing plants inoculated with *Mmag* for 10 d, yellow spots were uredinia. **b** Uredial density statistics. **c** Relative transcript levels of the *Mmag ITS* gene 10 d after inoculation. WT: Wild type 84 K poplar. V147: Silenced lines; OE 1, 5 7: Overexpressing lines. All data are presented as the means ± SD of three biologic replicates. Different letters above the bars indicate significant differences (*P* < 0.05)

To investigate the rust resistance of *PagMYB147-*silenced and OE plants, all plants were inoculated with *Mmag* to assess disease phenotypes and uredial density. The results demonstrated that *PagMYB147*-OE plants exhibited significantly enhanced resistance to *Mmag* inoculation compared to both WT and silenced plants (Fig. 9a). OE plants developed lower uredinia density with 20–27 uredinia/cm^2^, whereas silenced plants exhibited a significant (*P* < 0.05) increase in uredinia density with 68 uredinia/cm^2^ (Fig. 9b). Additionally, the biomass of *Mmag*, as indicated by the transcription level of the *ITS* gene, was measured in *PagMYB147*-silenced and OE plants at 10 d post-inoculation (dpi) using qRT-PCR. The results revealed that, after *Mmag* inoculation, the relative expression levels of *ITS* in *PagMYB147*-OE plants significantly (*P* < 0.05) decreased to 0.43–0.57 times that of WT plants. In contrast, the relative expression level of *ITS* in *PagMYB147*-silenced plants significantly (*P* < 0.05) increased to 2.20 times that of WT plants (Fig. 9c). These findings indicate that PagMYB147 positively contributes to poplar resistance to rust fungus *Mmag*.

### Analyses of physiologic traits

**Fig. 10:**
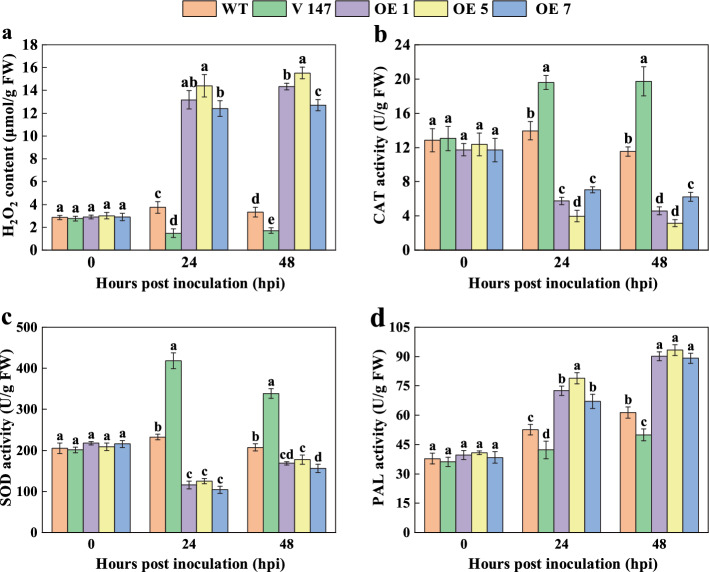
Physiologic and biochemical analysis of *Mmag* before and after inoculation in the WT, *PagMYB147*-silenced, and -overexpressing poplar plants. **a** H_2_O_2_ content. **b** CAT activity. **c** SOD activity. **d** PAL activity. All data are presented as the means ± SD of three biologic replicates. Different letters above the bars indicate significant differences (*P* < 0.05)

To investigate the effects of *PagMYB147-*silenced and OE on the physiologic status of leaves, the H_2_O_2_ content, CAT activity, SOD activity, and PAL activity of the leaves were also analyzed. Before inoculation, there were no significant changes in the H_2_O_2_ content and enzyme activities of CAT, SOD, and PAL in all plants (Fig. 10a–d). However, at 24 hpi and 48 hpi, the OE plants exhibited a significant (*P* < 0.05) increase in H_2_O_2_ content compared to WT plants, while the silenced plants showed a noteworthy decrease. Moreover, at the same time points, the OE plants showed significantly (*P* < 0.05) reduced CAT and SOD activities compared to WT plants, whereas the silenced plants displayed a significant (*P* < 0.05) increase. In addition, at 24 hpi and 48 hpi, the OE plants exhibited significantly (*P* < 0.05) increased PAL activity compared to WT plants, while the silenced plants displayed a notable decrease. Overall, these findings suggest that PagMYB147 is involved in the regulation of ROS levels and defense enzyme activities.

### Expression analyses of defense-related genes

**Fig. 11:**
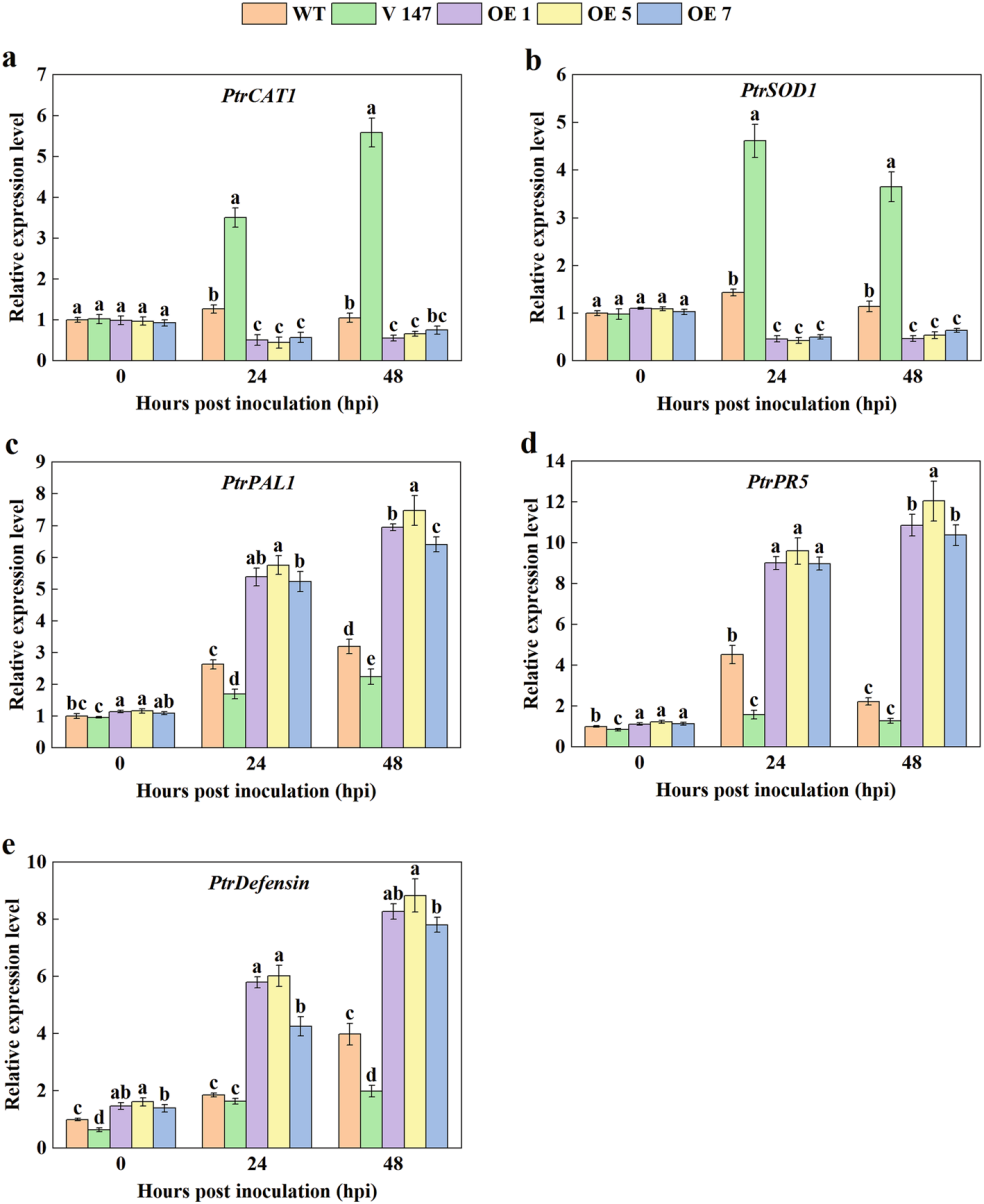
Relative expression levels of defense-related genes in the WT, *PagMYB147*-silenced, and -overexpressing poplar plants before and after inoculation. **a**-**e** Relative transcript levels of *PtrCAT1, PtrSOD1*, *PtrPAL1, PtrPR5*, and *PtrDefensin*. All data are presented as the means ± SD of three biologic replicates. Different letters above the bars indicate significant differences (*P* < 0.05)

To investigate the molecular regulatory role of PagMYB147, we evaluated the transcription levels of defense genes, including *PtrPAL1*, ROS scavenging genes (*PtrCAT1* and *PtrSOD1*), and SA/MeJA signaling pathway genes (*PtrPR5* and *PtrDefensin*). After *Mmag* inoculation, compared to the WT plants, the expression levels of *PtrCAT1* and *PtrSOD1* were significantly (*P* < 0.05) decreased in the OE plants but significantly (*P* < 0.05) increased in the silenced plants (Fig. 11a–b). In addition, the transcription levels of *PtrPAL1*, *PtrPR5*, and *PtrDefensin* were significantly (*P* < 0.05) increased in all plants after inoculation *Mmag*, but significantly (*P* < 0.05) slow in the silenced plants (Fig. 11c–e). Taken together, these findings indicate that PagMYB147 participates in poplar response to leaf rust fungus by directly or indirectly regulating the expression of ROS scavenging and certain defense-related genes.

## Discussion

The role of R2R3-MYB TFs is crucial in plants responses to biotic stresses (Seo and Park 2010; Mabuchi et al. 2018; Zhu et al. 2018). Previous studies identified 192 R2R3-MYB poplar genes from the Joint Genome Institute (JGI) Ptri 1.1 version database, and 196 or 207 R2R3-MYB poplar genes from the Ptri 3.0 version database (Wilkins et al. 2008; Zhao et al. 2020; Yang et al. 2021). With the advances in genomic information, we can now more accurately identify members of gene families. In our study, we identified 191 R2R3-MYB genes in the *P. trichocarpa* genome, categorizing them into 33 subgroups. The primary mechanisms driving gene family expansion are tandem and segmental duplications (Li et al. 2022). Specifically, we detected 12 tandem repeats on five chromosomes and 148 segmental repeats from 158 genes on 19 chromosomes in the *P. trichocarpa* genome. These findings highlight the significance of segmental duplications in the R2R3-MYB gene family expansion in *P. trichocarpa*. Moreover, our results showed that duplicated TFs have equivalent structures and motif compositions, as reported elsewhere (Zhao et al. 2020; Zhu et al. 2023). We found that 51 R2R3-MYBs in *P. trichocarpa* exhibit synteny with both *O. sativa* and *A. thaliana*, indicating that they had possibly evolved before the monocot–dicot split, as previously reported (Li et al. 2022; Wang et al. 2022). Consequently, our results support the notion that expansion of the *P. trichocarpa* R2R3-MYB gene subfamily may potentially result in the functional divergence of these genes, thereby facilitating plant adaptation to diverse environmental conditions.

R2R3-MYB genes have been shown to respond to the invasion of various pathogens in different plant species (Liu et al. 2015, 2021; Wang et al. 2017; Zhu et al. 2022; Ding et al. 2023). After inoculation with *M. larici-populina* E4, 34 R2R3-MYB genes showed significant differences in expression between tolerant and intolerant poplars (Chen et al. 2022). In this study, we identified 27 R2R3-MYB DEGs at 24 hpi and 26 R2R3-MYB DEGs at 144 hpi in poplar in response to *Mmag*, indicating the widespread involvement of R2R3-MYBs in response to rust infection. Among these, the *MYB147* gene was the most up-regulated in response to *Mmag*. Its silencing led to increased susceptibility, while overexpression enhanced resistance, suggesting a positive regulatory role in *Mmag* resistance. This is supported by studies showing increased resistance to the overexpression of *GhMYB108*, *TaMYB391* and *MYB115* (Cheng et al. 2016; James et al. 2017; Hawku et al. 2022). Plants generate ROS, including H_2_O_2_, to limit pathogen spread when attacked (Fichman et al. 2020). Overexpression of *PagMYB147* resulted in higher H_2_O_2_ levels post-*Mmag* inoculation, whereas silenced plants exhibited lower levels. Furthermore, plants possess a complex enzymatic antioxidant defense system, including CAT and SOD enzymes, which play a key role in regulating ROS homeostasis (Li et al. 2015a, b; Mabuchi et al. 2018). We observed that the activities of CAT and SOD were lower in *PagMYB147*-OE plants but higher in *PagMYB147*-silenced plants compared to WT plants. Furthermore, *PtrCAT1* and *PtrSOD1* gene expression corresponded with the enzymatic activity change, suggesting that PagMYB147 enhances rust resistance by positively regulating the ROS signaling pathway, the same did in wheat stripe rust resistance by overexpressing *TaMYB391* and *TaMYB29* through increasing the accumulation of ROS (Zhu et al. 2021; Hawku et al. 2022). The PAL enzyme, a key component of the phenylpropane pathway, plays a crucial role in plant defense against pathogens (He et al. 2019). Studies on *O. sativa* have shown that OsMYB30, OsMYB55, and OsMYB110 specifically activate *PAL* gene expression, contributing to pathogen resistance (Kishi-Kaboshi et al. 2018). Our study revealed that PagMYB147 can enhance PAL enzyme activity, thereby improving disease resistance. Li et al. (2014) also confirmed that the enhancement of resistance against *Alternaria alternata* in *SpMYB*-OE transgenic plants attributed to PAL accumulation. Moreover, R2R3-MYB TFs such as PacMYBA and MdMYB73 have been identified as regulators of genes associated with SA and JA signaling pathways, thereby regulating resistance to pathogens (Shen et al. 2017; Gu et al. 2020). Overexpression of *VqMYB154* not only enhances *Arabidopsis* resistance to *Uncinula necator* but also upregulates genes linked to the SA and JA/ET signaling pathways (Jiang et al. 2021). In this study, *PagMYB147* was found to improve plant resistance to rust fungus by positively modulating genes associated with the SA and JA pathways, indicating a beneficial role of PagMYB147 in enhancing rust resistance in poplar through collaboration with SA and JA signaling pathways.

**Fig. 12:**
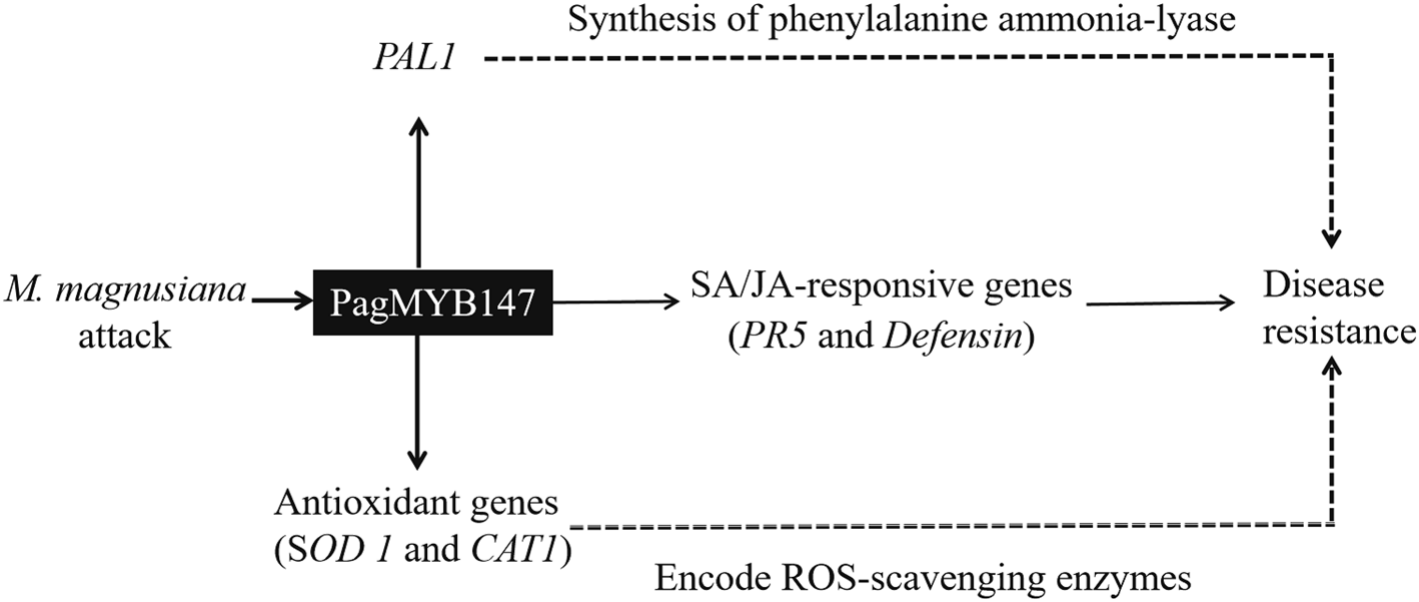
Hypothetical model of PagMYB147 regulation in rust resistance. The solid line denotes potential direct or indirect regulatory effects. The dotted line indicates this mode of regulation has been already confirmed

R2R3-MYB TFs regulate the expression of defense-related genes by binding to specific *cis*-acting elements in the promoter region (Cheng et al. 2016; Liu et al. 2021). Our findings suggest that PagMYB147 affects the transcription of five defense genes (*PtrSOD1*, *PtrCAT1*, *PtrPAL1*, *PtrPR5*, and *PtrDefensin*). We hypothesize that PagMYB147 may interact with *cis*-acting elements like MYB, MBS or MRE elements found in the promoter sequences of these defense genes (Table S8). Consequently, we speculate that PagMYB147 could regulate rust resistance by modulating the expression levels of these genes, directly or indirectly. However, further studies are needed to confirm whether PagMYB147 exerts its function through binding to these targets. Based on our data, we have proposed a hypothetical model to elucidate the function of PagMYB147 (Fig. 12). Our future research will focus on identifying the specific target genes regulated by PagMYB147, validating its function through gene editing, and investigating the associated signaling pathways. These investigations aim to provide deeper insights into the molecular signaling network of PagMYB147 in response to biotic stress.

## Conclusion

In total, 191 R2R3-MYB TFs were identified in the *P. trichocarpa* genome and were classified into 33 subgroups. Twelve tandem duplication events and 148 segmental duplication events were detected. The promoter sequences contain numerous *cis*-regulatory elements associated with responsiveness to plant stress and phytohormones. Through RNA-Seq data analysis, we identified multiple R2R3-MYB genes responsive to *Mmag*. Among them, nucleus-located *PagMYB147* was the most significantly up-regulated R2R3-MYB gene by *Mmag* infection. Silencing of *PagMYB147* in poplar reduced ROS accumulation, and down-regulate SA and JA-related genes, thereby reducing resistance against *Mmag*. Conversely, overexpression of *PagMYB147* showed the opposite ROS accumulation, PAL enzymes activity, SA and JA-related gene expressions, and thus improved *Mmag* resistance. All our results suggested that PagMYB147 positively regulates poplar resistance against *Mmag* through ROS homeostasis, SA and JA signaling pathway.

### Supplementary Information

Below is the link to the electronic supplementary material.

Supplementary file1 (DOC 22 KB)

Supplementary file2 (DOC 47 KB)

Supplementary file3 (DOC 17 KB)

Supplementary file4 (DOC 24 KB)

Supplementary file5 (DOC 24 KB)

Supplementary file6 (DOC 26 KB)

Supplementary file7 (DOC 17 KB)

Supplementary file8 (DOC 14 KB)

Supplementary file9 (EPS 2445 KB)

Supplementary file10 (EPS 13815 KB)

Supplementary file11 (EPS 13937 KB)

Supplementary file12 (EPS 1191 KB)

## Data Availability

The datasets generated during and/or analyzed during the current study are available from the corresponding author on reasonable request.

## Abbreviations

CAT: Catalase
DEG: Differentially expressed gene
Hpi: Hours post-inoculation
MeJA: Methyl jasmonate
*Mmag*: *Melampsora magnusiana*
OE: Overexpressing
PAL: Phenylalanine ammonia-lyase
ROS: Reactive oxygen species
SA: Salicylic acid
SOD: Superoxide dismutase
TF: Transcription factor
