# Supplementary material for: Genome-wide analysis of R2R3-MYB transcription factors in poplar and functional validation of PagMYB147 in defense against Melampsora magnusiana
Source: Planta. 2024 Jul 6;260(2):47. doi: 10.1007/s00425-024-04458-3 (PMC11227472; doi:10.1007/s00425-024-04458-3)
Supplement: Supplementary file 1 — Supplementary file1 (DOC 22 KB) [file 425_2024_4458_MOESM1_ESM.doc]

**Table S1. Primers used in this study**

| Name | Sequence (5' to 3') | Description and purpose |
| --- | --- | --- |
| *PtrMYB021* | GCTGTGAGAAGATGGGATTGA | Forward primer for qRT-PCR |
|  | TGTGCGTCCTGGTAATCTTG | Reverse primer for qRT-PCR |
| *PtrMYB025* | CTCAATGGACGCACCGATAA | Forward primer for qRT-PCR |
|  | GTGGTCTGTAAACCGGAGATG | Reverse primer for qRT-PCR |
| *PtrMYB026* | CTTCCGGGTCGTACTGATAATG | Forward primer for qRT-PCR |
|  | CACAAACTGCACCACCAATC | Reverse primer for qRT-PCR |
| *PtrMYB047* | TCCACGCAAGAAGATCAGAAG | Forward primer for qRT-PCR |
|  | TATTGCCAAGGAGAGCATGTAG | Reverse primer for qRT-PCR |
| *PtrMYB051* | CAGGCTTGTGAGAAAGGAAGAA | Forward primer for qRT-PCR |
|  | TCTGTTCTCTGAGGGAGGTATG | Reverse primer for qRT-PCR |
| *PtrMYB058* | CAACGAAGAAGCCTCGTATCT | Forward primer for qRT-PCR |
|  | TCTGGTTTGTTTACCTCCTCTG | Reverse primer for qRT-PCR |
| *PtrMYB073* | CCCTGCTCAACATCTCCTATTC | Forward primer for qRT-PCR |
|  | GCACTGAGAGACCCTTGTTATC | Reverse primer for qRT-PCR |
| *PtrMYB077* | CTGACGTTAAGCGTGGTAACT | Forward primer for qRT-PCR |
|  | GAAGTTGAGGAGGATCCCTTTG | Reverse primer for qRT-PCR |
| *PtrMYB120* | GCACAGCCTTCCAGGATATAAT | Forward primer for qRT-PCR |
|  | TTGCTCACGATGCTCCATAC | Reverse primer for qRT-PCR |
| *PtrMYB128* | CCATCCGTGTCAACCTCTATG | Forward primer for qRT-PCR |
|  | GGATTTCCCACAATCAGCTAAAC | Reverse primer for qRT-PCR |
| *PtrMYB143* | GCAGAACGGACAACGAGATAA | Forward primer for qRT-PCR |
|  | TCAATCTGGCTGGAATGGTAAG | Reverse primer for qRT-PCR |
| *PtrMYB147* | CCCAAAGACCAGCAGTAGTT | Forward primer for qRT-PCR |
|  | TGAAAGGGACTTGTAGGGTATTC | Reverse primer for qRT-PCR |
| *PtrMYB149* | TGGACAGTGGAGGAAGATAGA | Forward primer for qRT-PCR |
|  | GGAATCCGACCAGCAATTAGA | Reverse primer for qRT-PCR |
| *PtrMYB161* | GTCCTTGAGTCTCCAACATCTAC | Forward primer for qRT-PCR |
|  | CATCGTCTGACAAACTCCTCTC | Reverse primer for qRT-PCR |
| *PagMYB147* | GGGGTACCATGAGCGAGGACATCAT | Forward primer for amplify the ORF regions |
|  | CGGGATCCTTAATTTTCCTTTCCTTGC | Reverse primer for amplify the ORF regions |
| *PagMYB147-*EGFP | AACACGGGGGACTTTGCAACATGAGCG  AGGACATCATCATGTCTTCTTTAAAAAAG | Forward primer for the subcellular localization |
|  | CTCCCTGAAGCGGCCGCTGTACA  ATTTTCCTTTCCTTGCAAGTTC | Reverse primer for the subcellular localization |
| TRV2*-PagMYB147* | CGGGATCCATGAGCGAGGACATCAT | Forward primer for VIGS of *PagMYB147* |
|  | GGGGTACCCCTGGCAAATGTTGTGC | Reverse primer for VIGS of *PagMYB147* |
| *PagMYB147* | CCCAAAGACCAGCAGTAGTT | Forward primer for qRT-PCR |
|  | TTCTCCAAGCACTGGCATAG | Reverse primer for qRT-PCR |
| *35S:PagMYB147* | TTCATTTGGAGAGAACACGGGGGAC | Forward primer for transgenic positive plant |
|  | CATGAACTTGTACCAAGCTCTG | Reverse primer for transgenic positive plant |
| *PtrSOD1* | CAAGCCGCAATAGCAGCCAT | Forward primer for qRT-PCR |
|  | CCGTGGAATGCAGAGTGAAG | Reverse primer for qRT-PCR |
| *PtrCAT1* | CTGCCAGTTCTTTCAACGCC | Forward primer for qRT-PCR |
|  | AAACCCTTAGCACTGGCTCC | Reverse primer for qRT-PCR |
| *PtrPAL1* | AGAACCGTCGATGGAATCAC | Forward primer for qRT-PCR |
|  | GGTCTCTCTGGCTGCTATGG | Reverse primer for qRT-PCR |
| *PtrPR5* | GAATGCCGGAACAACACAAG | Forward primer for qRT-PCR |
|  | GTTGCCTGATACTGGACTGAA | Reverse primer for qRT-PCR |
| *PtrDefensin* | TGGCTAGCTTCACCAGTCTT | Forward primer for qRT-PCR |
|  | ATGCTCCTGTTCCCTGGTTT | Reverse primer for qRT-PCR |
| *PagActin* | GAAGTCCTCTTCCAGCCTTCTC | Forward primer for qRT-PCR |
|  | CTTGATCTTCATGCTGCTTGGG | Reverse primer for qRT-PCR |
| *Mmag-ITS* | TCACGCCTCGCTTCAAATAG | Forward primer for qRT-PCR |
|  | TGTTTAAGTTCAGCGGGTAGTC | Reverse primer for qRT-PCR |
| *ELF1α* | CGAGACTCCCAAATACTTCGTT | Forward primer for qRT-PCR |
|  | GTTCACGAGTTTGACCATCCTT | Reverse primer for qRT-PCR |
