# Supplementary material for: Genome-wide analysis of R2R3-MYB transcription factors in poplar and functional validation of PagMYB147 in defense against Melampsora magnusiana
Source: Planta. 2024 Jul 6;260(2):47. doi: 10.1007/s00425-024-04458-3 (PMC11227472; doi:10.1007/s00425-024-04458-3)
Supplement: Supplementary file 2 — Supplementary file2 (DOC 47 KB) [file 425_2024_4458_MOESM2_ESM.doc]

**Table S2.** **The length distribution and physicochemical properties of *P. trichocarpa* R2R3-MYB proteins**

| Gene name | Gene ID | Peptide length (aa) | Molecular weight (kDa) | Isoelectric point | GRAVY score | Instability index | Signal peptides | Subcellular localization |
| --- | --- | --- | --- | --- | --- | --- | --- | --- |
| PtrMYB001 | Potri.001G005100.1 | 316 | 35.26 | 7.68 | -0.656 | 42.34 | No | Nucleus |
| PtrMYB002 | Potri.001G036000.1 | 568 | 62.68 | 5.15 | -0.642 | 61.91 | No | Nucleus |
| PtrMYB003 | Potri.001G075400.1 | 340 | 38.47 | 6.46 | -0.607 | 48.68 | No | Nucleus |
| PtrMYB004 | Potri.001G086700.1 | 267 | 29.95 | 9.33 | -0.8 | 40.89 | No | Nucleus |
| PtrMYB005 | Potri.001G099800.1 | 309 | 34.92 | 5.39 | -0.56 | 58.43 | No | Nucleus |
| PtrMYB006 | Potri.001G118800.1 | 270 | 30.66 | 5.24 | -0.718 | 56.1 | No | Nucleus |
| PtrMYB007 | Potri.001G139900.1 | 336 | 37.74 | 6.19 | -0.712 | 34.25 | No | Nucleus |
| PtrMYB008 | Potri.001G169600.1 | 194 | 22.54 | 8.9 | -0.92 | 60.33 | No | Nucleus |
| PtrMYB009 | Potri.001G197000.1 | 367 | 40.74 | 6.32 | -0.462 | 66.89 | No | Nucleus |
| PtrMYB010 | Potri.001G224500.3 | 529 | 57.88 | 5.91 | -0.591 | 52.07 | No | Nucleus |
| PtrMYB011 | Potri.001G235500.1 | 241 | 27.31 | 7.62 | -0.727 | 53.83 | No | Nucleus |
| PtrMYB012 | Potri.001G250000.1 | 471 | 52.24 | 6.87 | -0.893 | 46.43 | No | Nucleus |
| PtrMYB013 | Potri.001G258700.1 | 333 | 37.03 | 5.41 | -0.688 | 52.37 | No | Nucleus |
| PtrMYB014 | Potri.001G267300.1 | 327 | 37.02 | 5.83 | -0.645 | 58.2 | No | Nucleus |
| PtrMYB015 | Potri.001G300200.1 | 244 | 26.53 | 9.22 | -0.407 | 36.68 | No | Nucleus |
| PtrMYB016 | Potri.001G336700.1 | 282 | 32.68 | 5.31 | -0.829 | 47.64 | No | Nucleus |
| PtrMYB017 | Potri.001G346600.4 | 190 | 21.66 | 6.52 | -0.822 | 48.66 | No | Nucleus |
| PtrMYB018 | Potri.001G347200.1 | 450 | 50.92 | 5.71 | -0.814 | 47.63 | No | Nucleus |
| PtrMYB019 | Potri.001G408700.1 | 351 | 39.51 | 7.97 | -0.565 | 52.5 | No | Nucleus |
| PtrMYB020 | Potri.001G470500.1 | 327 | 36.42 | 6.55 | -0.552 | 37.31 | No | Nucleus |
| PtrMYB021 | Potri.002G038500.1 | 272 | 31 | 5.51 | -0.549 | 55.14 | No | Nucleus |
| PtrMYB022 | Potri.002G073500.1 | 333 | 38.05 | 9.48 | -1.017 | 68.26 | No | Nucleus |
| PtrMYB023 | Potri.002G096800.1 | 336 | 37.29 | 6.09 | -0.483 | 50.43 | No | Nucleus |
| PtrMYB024 | Potri.002G113700.2 | 345 | 38.63 | 5.93 | -0.698 | 51.95 | No | Nucleus |
| PtrMYB025 | Potri.002G122600.1 | 307 | 33.27 | 8.73 | -0.55 | 58.98 | No | Nucleus |
| PtrMYB026 | Potri.002G128900.1 | 237 | 26.31 | 6.91 | -0.647 | 52.42 | No | Nucleus |
| PtrMYB027 | Potri.002G140900.1 | 308 | 34.85 | 5.78 | -0.625 | 69.76 | No | Nucleus |
| PtrMYB028 | Potri.002G157600.1 | 315 | 35.03 | 7.55 | -0.524 | 61.38 | No | Nucleus |
| PtrMYB029 | Potri.002G173900.1 | 285 | 32.18 | 9.21 | -0.81 | 49.12 | No | Nucleus |
| PtrMYB030 | Potri.002G185900.1 | 464 | 51.82 | 6.68 | -0.793 | 53.39 | No | Nucleus |
| PtrMYB031 | Potri.002G191800.2 | 294 | 33.42 | 5.3 | -0.714 | 58.91 | No | Nucleus |
| PtrMYB032 | Potri.002G198100.1 | 402 | 44.34 | 5.09 | -0.664 | 55.78 | No | Nucleus |
| PtrMYB033 | Potri.002G228700.1 | 496 | 55.13 | 7.13 | -0.78 | 51.57 | No | Nucleus |
| PtrMYB034 | Potri.003G064600.1 | 197 | 22.85 | 9.45 | -0.93 | 48.49 | No | Nucleus |
| PtrMYB035 | Potri.003G079100.1 | 180 | 20.52 | 8.65 | -0.767 | 53.17 | No | Nucleus |
| PtrMYB036 | Potri.003G094200.2 | 339 | 37.84 | 6.46 | -0.601 | 41.05 | No | Nucleus |
| PtrMYB037 | Potri.003G114100.1 | 270 | 30.62 | 5.1 | -0.678 | 52.73 | No | Nucleus |
| PtrMYB038 | Potri.003G132000.2 | 311 | 35.02 | 5.53 | -0.546 | 57.11 | No | Nucleus |
| PtrMYB039 | Potri.003G144200.1 | 280 | 31.26 | 8.91 | -0.697 | 49.72 | No | Nucleus |
| PtrMYB040 | Potri.003G144300.1 | 272 | 30.76 | 9.11 | -0.824 | 50.88 | No | Nucleus |
| PtrMYB041 | Potri.003G155700.1 | 341 | 38.42 | 6.56 | -0.589 | 48.94 | No | Nucleus |
| PtrMYB042 | Potri.003G189700.7 | 568 | 62.1 | 5.06 | -0.617 | 54.48 | No | Nucleus |
| PtrMYB043 | Potri.003G219900.1 | 325 | 35.82 | 8.25 | -0.688 | 44 | No | Nucleus |
| PtrMYB044 | Potri.004G026600.1 | 254 | 28.86 | 8.68 | -0.539 | 47.55 | No | Nucleus |
| PtrMYB045 | Potri.004G033100.1 | 376 | 42.35 | 5.69 | -0.67 | 48.38 | No | Nucleus |
| PtrMYB046 | Potri.004G086300.1 | 319 | 35.82 | 5.09 | -0.696 | 55.85 | No | Nucleus |
| PtrMYB047 | Potri.004G088100.1 | 245 | 27.14 | 9.18 | -0.693 | 42.8 | No | Nucleus |
| PtrMYB048 | Potri.004G102600.1 | 361 | 41.75 | 9.22 | -0.946 | 59.34 | No | Nucleus |
| PtrMYB049 | Potri.004G115600.1 | 211 | 24.72 | 6.76 | -0.916 | 60.28 | No | Nucleus |
| PtrMYB050 | Potri.004G118000.1 | 212 | 24.81 | 7.02 | -0.865 | 62.25 | No | Nucleus |
| PtrMYB051 | Potri.004G126700.1 | 354 | 40.3 | 8.9 | -0.688 | 53.85 | No | Nucleus |
| PtrMYB052 | Potri.004G138000.1 | 319 | 35.8 | 8.45 | -0.749 | 51.62 | No | Nucleus |
| PtrMYB053 | Potri.004G174400.1 | 268 | 29.85 | 8.88 | -0.583 | 45.54 | No | Nucleus |
| PtrMYB054 | Potri.004G215100.3 | 337 | 37.77 | 6.66 | -0.747 | 54.6 | No | Nucleus |
| PtrMYB055 | Potri.005G001600.1 | 441 | 49.11 | 7.16 | -0.723 | 54.89 | No | Nucleus |
| PtrMYB056 | Potri.005G063200.1 | 425 | 48.37 | 9.03 | -0.547 | 58.61 | No | Nucleus |
| PtrMYB057 | Potri.005G074500.1 | 341 | 37.93 | 6.35 | -0.514 | 56.16 | No | Nucleus |
| PtrMYB058 | Potri.005G096600.1 | 367 | 41.81 | 6.14 | -0.934 | 48.05 | No | Nucleus |
| PtrMYB059 | Potri.005G112000.2 | 245 | 27.88 | 9.28 | -0.668 | 40.94 | No | Nucleus |
| PtrMYB060 | Potri.005G118500.1 | 427 | 49.45 | 5.78 | -0.802 | 58.18 | No | Nucleus |
| PtrMYB061 | Potri.005G142600.1 | 351 | 37.91 | 8.78 | -0.654 | 48.79 | No | Nucleus |
| PtrMYB062 | Potri.005G164900.1 | 337 | 37.87 | 5.57 | -0.586 | 45.87 | No | Nucleus |
| PtrMYB063 | Potri.005G186400.1 | 334 | 37.71 | 9.25 | -0.869 | 63.09 | No | Nucleus |
| PtrMYB064 | Potri.005G224100.1 | 273 | 31.16 | 5.16 | -0.624 | 55.46 | No | Nucleus |
| PtrMYB065 | Potri.006G066400.1 | 233 | 27.11 | 8.97 | -1.038 | 70.53 | No | Nucleus |
| PtrMYB066 | Potri.006G085900.1 | 369 | 42.67 | 9.36 | -0.952 | 64.15 | No | Nucleus |
| PtrMYB067 | Potri.006G122100.1 | 234 | 27.14 | 5.45 | -0.954 | 52 | No | Nucleus |
| PtrMYB068 | Potri.006G123400.1 | 333 | 36.89 | 6.53 | -0.615 | 51.54 | No | Nucleus |
| PtrMYB069 | Potri.006G170800.1 | 365 | 41.05 | 6.46 | -0.651 | 52.61 | No | Nucleus |
| PtrMYB070 | Potri.006G221200.1 | 197 | 22.72 | 9.1 | -0.92 | 64.71 | No | Nucleus |
| PtrMYB071 | Potri.006G221500.1 | 267 | 30.03 | 5.46 | -0.66 | 51.42 | No | Nucleus |
| PtrMYB072 | Potri.006G221800.1 | 288 | 32.44 | 8.41 | -0.748 | 56.96 | No | Nucleus |
| PtrMYB073 | Potri.006G234200.1 | 302 | 32.91 | 5.63 | -0.326 | 53.42 | No | Nucleus |
| PtrMYB074 | Potri.006G275900.2 | 303 | 33.95 | 5.59 | -0.683 | 45.28 | No | Nucleus |
| PtrMYB075 | Potri.007G007900.1 | 343 | 38.15 | 6.33 | -0.574 | 60.46 | No | Nucleus |
| PtrMYB076 | Potri.007G048900.5 | 330 | 35.75 | 8.53 | -0.627 | 50.22 | No | Nucleus |
| PtrMYB077 | Potri.007G067600.1 | 367 | 41.25 | 5.82 | -0.918 | 47.47 | No | Nucleus |
| PtrMYB078 | Potri.007G093900.1 | 341 | 37.79 | 6.35 | -0.461 | 56.14 | No | Nucleus |
| PtrMYB079 | Potri.007G106100.3 | 283 | 32.7 | 9.74 | -0.842 | 60.7 | No | Nucleus |
| PtrMYB080 | Potri.007G134500.2 | 247 | 28.89 | 8.63 | -0.916 | 66.71 | No | Nucleus |
| PtrMYB081 | Potri.008G062700.1 | 412 | 44.61 | 5.65 | -0.696 | 53.86 | No | Nucleus |
| PtrMYB082 | Potri.008G070900.1 | 259 | 28.35 | 8.58 | -0.599 | 45.84 | No | Nucleus |
| PtrMYB083 | Potri.008G081600.2 | 338 | 38.05 | 6.45 | -0.542 | 60.91 | No | Nucleus |
| PtrMYB084 | Potri.008G088000.1 | 448 | 49.62 | 7.17 | -0.699 | 55.68 | No | Nucleus |
| PtrMYB085 | Potri.008G089200.1 | 382 | 41.96 | 7.62 | -0.485 | 41.1 | No | Nucleus |
| PtrMYB086 | Potri.008G089700.1 | 381 | 41.91 | 8.34 | -0.52 | 45.73 | No | Nucleus |
| PtrMYB087 | Potri.008G101400.1 | 322 | 36.38 | 6.51 | -0.704 | 43.2 | No | Nucleus |
| PtrMYB088 | Potri.008G122100.1 | 314 | 35.95 | 6.2 | -0.727 | 60.64 | No | Nucleus |
| PtrMYB089 | Potri.008G128500.1 | 221 | 24.95 | 9.08 | -0.742 | 50.09 | No | Nucleus |
| PtrMYB090 | Potri.008G148400.5 | 480 | 54.25 | 7.96 | -0.734 | 62.36 | No | Nucleus |
| PtrMYB091 | Potri.008G166700.1 | 287 | 32.78 | 5.72 | -0.732 | 59.59 | No | Nucleus |
| PtrMYB092 | Potri.008G173400.1 | 264 | 30.65 | 9.19 | -0.795 | 60.5 | No | Nucleus |
| PtrMYB093 | Potri.008G180800.2 | 279 | 31.8 | 6.09 | -0.499 | 51.93 | No | Nucleus |
| PtrMYB094 | Potri.009G007100.1 | 338 | 38.11 | 6.53 | -0.736 | 50.56 | No | Nucleus |
| PtrMYB095 | Potri.009G018700.1 | 516 | 56.63 | 5.05 | -0.576 | 52.94 | No | Nucleus |
| PtrMYB096 | Potri.009G027300.1 | 243 | 27.52 | 6.85 | -0.746 | 59.95 | No | Nucleus |
| PtrMYB097 | Potri.009G044100.1 | 484 | 53.53 | 5.5 | -0.829 | 52.19 | No | Nucleus |
| PtrMYB098 | Potri.009G053900.1 | 332 | 37.09 | 5.26 | -0.619 | 62.12 | No | Nucleus |
| PtrMYB099 | Potri.009G061500.1 | 333 | 37.71 | 5.46 | -0.722 | 51.38 | No | Nucleus |
| PtrMYB100 | Potri.009G096000.1 | 245 | 26.81 | 6.33 | -0.549 | 40.46 | No | Nucleus |
| PtrMYB101 | Potri.009G134000.1 | 271 | 30.22 | 8.72 | -0.557 | 45.94 | No | Nucleus |
| PtrMYB102 | Potri.010G004300.1 | 352 | 39.95 | 8.05 | -0.754 | 56.66 | No | Nucleus |
| PtrMYB103 | Potri.010G064000.1 | 261 | 30.36 | 9.15 | -0.796 | 68.94 | No | Nucleus |
| PtrMYB104 | Potri.010G093000.1 | 483 | 54.38 | 7.17 | -0.69 | 56.2 | No | Nucleus |
| PtrMYB105 | Potri.010G114000.2 | 226 | 25.6 | 9.1 | -0.853 | 53.34 | No | Nucleus |
| PtrMYB106 | Potri.010G123000.1 | 319 | 36.14 | 6.34 | -0.701 | 65.54 | No | Nucleus |
| PtrMYB107 | Potri.010G141000.1 | 364 | 40.66 | 8.51 | -0.792 | 47.53 | No | Nucleus |
| PtrMYB108 | Potri.010G149900.1 | 317 | 35.97 | 5.68 | -0.713 | 50.62 | No | Nucleus |
| PtrMYB109 | Potri.010G165700.1 | 382 | 41.71 | 6.32 | -0.518 | 46.66 | No | Nucleus |
| PtrMYB110 | Potri.010G167500.2 | 449 | 49.85 | 6.64 | -0.641 | 61.14 | No | Nucleus |
| PtrMYB111 | Potri.010G174500.1 | 337 | 37.89 | 7.95 | -0.541 | 57.23 | No | Nucleus |
| PtrMYB112 | Potri.010G195000.1 | 415 | 44.77 | 5.58 | -0.708 | 48.4 | No | Nucleus |
| PtrMYB113 | Potri.011G040200.1 | 275 | 31.57 | 5.92 | -0.818 | 49.33 | No | Nucleus |
| PtrMYB114 | Potri.011G040300.1 | 273 | 31.63 | 5.71 | -0.789 | 52.93 | No | Nucleus |
| PtrMYB115 | Potri.011G040400.1 | 308 | 35.64 | 4.92 | -0.759 | 48.68 | No | Nucleus |
| PtrMYB116 | Potri.011G041600.1 | 375 | 42.24 | 5.93 | -0.681 | 51.7 | No | Nucleus |
| PtrMYB117 | Potri.011G125900.1 | 351 | 39.49 | 6.63 | -0.64 | 58.03 | No | Nucleus |
| PtrMYB118 | Potri.011G167600.1 | 327 | 36.21 | 6.79 | -0.577 | 36.21 | No | Nucleus |
| PtrMYB119 | Potri.012G039400.1 | 243 | 28.76 | 7.17 | -1.067 | 67.78 | No | Nucleus |
| PtrMYB120 | Potri.012G055600.1 | 327 | 37.25 | 7.15 | -0.672 | 66.71 | No | Nucleus |
| PtrMYB121 | Potri.012G072500.1 | 368 | 41.35 | 5.68 | -0.507 | 54.39 | No | Nucleus |
| PtrMYB122 | Potri.012G080400.1 | 216 | 24.63 | 8.68 | -0.745 | 49.42 | No | Nucleus |
| PtrMYB123 | Potri.012G082000.1 | 447 | 50.15 | 6.44 | -0.723 | 51.99 | No | Nucleus |
| PtrMYB124 | Potri.012G084100.1 | 370 | 41.64 | 6.37 | -0.575 | 53.36 | No | Nucleus |
| PtrMYB125 | Potri.012G127700.1 | 276 | 30.75 | 5.39 | -0.686 | 57.84 | No | Nucleus |
| PtrMYB126 | Potri.012G140500.1 | 293 | 33.38 | 6.8 | -0.817 | 65.69 | No | Nucleus |
| PtrMYB127 | Potri.012G140700.2 | 298 | 33.67 | 6.26 | -0.642 | 50.95 | No | Nucleus |
| PtrMYB128 | Potri.013G001000.1 | 446 | 49.42 | 7.56 | -0.597 | 51.47 | No | Nucleus |
| PtrMYB129 | Potri.013G056400.1 | 304 | 34.27 | 6.67 | -0.793 | 53.83 | No | Nucleus |
| PtrMYB130 | Potri.013G056500.1 | 308 | 34.9 | 8.17 | -0.771 | 53.18 | No | Nucleus |
| PtrMYB131 | Potri.013G067000.1 | 394 | 44.07 | 8.52 | -0.778 | 50.29 | No | Nucleus |
| PtrMYB132 | Potri.013G067500.1 | 323 | 36.5 | 6.13 | -0.769 | 45.66 | No | Nucleus |
| PtrMYB133 | Potri.013G109300.1 | 301 | 34.43 | 8.92 | -0.969 | 51.88 | No | Nucleus |
| PtrMYB134 | Potri.013G148600.1 | 362 | 40.3 | 5.28 | -0.508 | 50.29 | No | Nucleus |
| PtrMYB135 | Potri.013G149100.1 | 265 | 30.49 | 4.85 | -0.935 | 70.7 | No | Nucleus |
| PtrMYB136 | Potri.013G149200.1 | 256 | 29.41 | 4.72 | -0.796 | 58.54 | No | Nucleus |
| PtrMYB137 | Potri.014G022500.1 | 311 | 33.72 | 9.02 | -0.487 | 57.83 | No | Nucleus |
| PtrMYB138 | Potri.014G035100.1 | 248 | 27.41 | 8.51 | -0.743 | 58.44 | No | Nucleus |
| PtrMYB139 | Potri.014G054700.1 | 303 | 34.36 | 5.34 | -0.619 | 71.67 | No | Nucleus |
| PtrMYB140 | Potri.014G081200.1 | 316 | 34.52 | 6.61 | -0.486 | 61.54 | No | Nucleus |
| PtrMYB141 | Potri.014G100800.1 | 270 | 30.74 | 9.63 | -0.812 | 54.64 | No | Nucleus |
| PtrMYB142 | Potri.014G111200.1 | 464 | 51.73 | 8.25 | -0.805 | 54.32 | No | Nucleus |
| PtrMYB143 | Potri.014G117000.1 | 287 | 32.68 | 6.06 | -0.802 | 54.15 | No | Nucleus |
| PtrMYB144 | Potri.014G122700.2 | 417 | 46.51 | 5.13 | -0.664 | 47.24 | No | Nucleus |
| PtrMYB145 | Potri.015G033600.1 | 236 | 28.11 | 7.71 | -0.929 | 65.61 | No | Nucleus |
| PtrMYB146 | Potri.015G041100.1 | 185 | 21.18 | 9.46 | -1.022 | 57.74 | No | Nucleus |
| PtrMYB147 | Potri.015G046200.1 | 332 | 37.74 | 6.26 | -0.669 | 72.65 | No | Nucleus |
| PtrMYB148 | Potri.015G067700.1 | 385 | 42.85 | 5.57 | -0.469 | 44.24 | No | Nucleus |
| PtrMYB149 | Potri.015G075600.2 | 212 | 24.37 | 8.94 | -0.662 | 48.4 | No | Nucleus |
| PtrMYB150 | Potri.015G077700.1 | 436 | 49.24 | 5.67 | -0.646 | 52 | No | Nucleus |
| PtrMYB151 | Potri.015G082700.1 | 371 | 41.91 | 6.01 | -0.613 | 51.5 | No | Nucleus |
| PtrMYB152 | Potri.015G129100.1 | 274 | 30.81 | 5.91 | -0.686 | 47.57 | No | Nucleus |
| PtrMYB153 | Potri.015G143400.2 | 295 | 33.48 | 6.27 | -0.771 | 60.6 | No | Nucleus |
| PtrMYB154 | Potri.015G143500.2 | 296 | 32.91 | 6.37 | -0.509 | 44.38 | No | Nucleus |
| PtrMYB155 | Potri.016G099200.1 | 331 | 36.8 | 7.06 | -0.663 | 53.77 | No | Nucleus |
| PtrMYB156 | Potri.017G017600.1 | 241 | 28.27 | 8.55 | -0.923 | 56.95 | No | Nucleus |
| PtrMYB157 | Potri.017G071500.1 | 190 | 21.69 | 6.31 | -0.813 | 56.08 | No | Nucleus |
| PtrMYB158 | Potri.017G075000.1 | 380 | 42.7 | 5.84 | -0.659 | 56.35 | No | Nucleus |
| PtrMYB159 | Potri.017G082500.1 | 322 | 35.73 | 6.91 | -0.674 | 51.68 | No | Nucleus |
| PtrMYB160 | Potri.017G085200.1 | 437 | 48.81 | 8.53 | -0.616 | 59.12 | No | Nucleus |
| PtrMYB161 | Potri.017G086300.3 | 401 | 43.72 | 6.28 | -0.694 | 50.48 | No | Nucleus |
| PtrMYB162 | Potri.017G099500.2 | 218 | 25.53 | 8.36 | -0.942 | 67.55 | No | Nucleus |
| PtrMYB163 | Potri.017G112300.1 | 363 | 41.32 | 9.31 | -0.847 | 59.17 | No | Nucleus |
| PtrMYB164 | Potri.017G125600.1 | 278 | 31.62 | 8.6 | -0.61 | 46.17 | No | Nucleus |
| PtrMYB165 | Potri.017G125700.1 | 268 | 30.26 | 8.9 | -0.592 | 41.65 | No | Nucleus |
| PtrMYB166 | Potri.017G125800.1 | 267 | 30.35 | 8.38 | -0.613 | 37.94 | No | Nucleus |
| PtrMYB167 | Potri.017G125900.1 | 258 | 29.22 | 8.72 | -0.447 | 42.97 | No | Nucleus |
| PtrMYB168 | Potri.017G126000.1 | 282 | 32.15 | 8.78 | -0.513 | 48.88 | No | Nucleus |
| PtrMYB169 | Potri.017G128900.1 | 244 | 27.23 | 8.99 | -0.759 | 44.55 | No | Nucleus |
| PtrMYB170 | Potri.017G130300.1 | 321 | 36.01 | 5.1 | -0.563 | 64.34 | No | Nucleus |
| PtrMYB171 | Potri.018G005300.1 | 391 | 43.47 | 5.67 | -0.82 | 46.12 | No | Nucleus |
| PtrMYB172 | Potri.018G049000.1 | 200 | 23.13 | 9.4 | -0.884 | 71.08 | No | Nucleus |
| PtrMYB173 | Potri.018G049401.1 | 296 | 33.25 | 8.53 | -0.761 | 55.37 | No | Nucleus |
| PtrMYB174 | Potri.018G049600.1 | 296 | 33.21 | 8.34 | -0.775 | 56.63 | No | Nucleus |
| PtrMYB175 | Potri.018G058800.4 | 257 | 28.6 | 9.16 | -0.282 | 60.89 | No | Nucleus |
| PtrMYB176 | Potri.018G095900.1 | 366 | 41.02 | 5.9 | -0.672 | 50.76 | No | Nucleus |
| PtrMYB177 | Potri.018G127700.1 | 230 | 26.32 | 7.71 | -1.066 | 66.16 | No | Nucleus |
| PtrMYB178 | Potri.019G018200.2 | 219 | 25.08 | 9.17 | -0.801 | 52.91 | No | Nucleus |
| PtrMYB179 | Potri.019G036160.1 | 309 | 34.61 | 9.25 | -0.691 | 53.94 | No | Nucleus |
| PtrMYB180 | Potri.019G036340.1 | 309 | 34.58 | 9.23 | -0.723 | 50.87 | No | Nucleus |
| PtrMYB181 | Potri.019G036400.1 | 309 | 34.7 | 9.12 | -0.748 | 53.51 | No | Nucleus |
| PtrMYB182 | Potri.019G040900.1 | 379 | 42.09 | 8.22 | -0.7 | 49.76 | No | Nucleus |
| PtrMYB183 | Potri.019G045900.1 | 325 | 37.05 | 5.96 | -0.886 | 49.6 | No | Nucleus |
| PtrMYB184 | Potri.019G050900.3 | 340 | 38.25 | 5.79 | -0.637 | 48.97 | No | Nucleus |
| PtrMYB185 | Potri.019G081500.1 | 298 | 33.73 | 8.93 | -0.872 | 53.77 | No | Nucleus |
| PtrMYB186 | Potri.019G118200.1 | 364 | 40.57 | 5.84 | -0.49 | 51.81 | No | Nucleus |
| PtrMYB187 | Potri.019G118700.1 | 262 | 30.28 | 4.9 | -0.96 | 72.05 | No | Nucleus |
| PtrMYB188 | Potri.019G118800.1 | 263 | 30.25 | 5.22 | -0.819 | 56.37 | No | Nucleus |
| PtrMYB189 | Potri.019G118900.1 | 250 | 28.4 | 4.79 | -0.722 | 58.73 | No | Nucleus |
| PtrMYB190 | Potri.T011400.1 | 318 | 35.57 | 8.8 | -0.688 | 46.45 | No | Nucleus |
| PtrMYB190.1 | Potri.T011525.1 | 318 | 35.57 | 8.8 | -0.688 | 46.45 | No | Nucleus |
