# Supplementary material for: Genome-wide analysis of R2R3-MYB transcription factors in poplar and functional validation of PagMYB147 in defense against Melampsora magnusiana
Source: Planta. 2024 Jul 6;260(2):47. doi: 10.1007/s00425-024-04458-3 (PMC11227472; doi:10.1007/s00425-024-04458-3)
Supplement: Supplementary file 3 — Supplementary file3 (DOC 17 KB) [file 425_2024_4458_MOESM3_ESM.doc]

**Table S3. Number of R2R3-MYB transcription factors in different subgroups**

| Subgroup | Number of *P. trichocarpa* R2R3-MYBs | Number of *A. thaliana* R2R3-MYBs | Function |
| --- | --- | --- | --- |
| S1 | 4 | 5 | Defense |
| S2 | 4 | 3 | Defense |
| S3 | 2 | 4 | Metabolism |
| S4 | 13 | 6 | Metabolism |
| S5 | 6 | 1 | Metabolism |
| S6 | 5 | 4 | Metabolism |
| S7 | 15 | 4 | Metabolism |
| S9 | 8 | 3 | Differentiation |
| S10 | 3 | 3 | Metabolism |
| S11 | 6 | 4 | Defense |
| S12 | 0 | 6 | Metabolism |
| S13 | 7 | 4 | Metabolism |
| S14 | 15 | 8 | Development |
| S15 | 2 | 3 | Differentiation |
| S16 | 13 | 8 | Development and metabolism |
| S18 | 8 | 8 | Development and defense |
| S19 | 2 | 3 | Development |
| S20 | 8 | 6 | Defense |
| S21 | 13 | 8 | Metabolism and development |
| S22 | 9 | 4 | Defense |
| S23 | 2 | 3 | Defense |
| S24 | 4 | 3 | Development |
| S25 | 10 | 10 | Differentiation |
| S26 | 2 | 2 | Development |
| S28 | 8 | 6 | Metabolism |
| N | 22 | 7 | - |
