# Supplementary material for: Genome-wide analysis of R2R3-MYB transcription factors in poplar and functional validation of PagMYB147 in defense against Melampsora magnusiana
Source: Planta. 2024 Jul 6;260(2):47. doi: 10.1007/s00425-024-04458-3 (PMC11227472; doi:10.1007/s00425-024-04458-3)
Supplement: Supplementary file 4 — Supplementary file4 (DOC 24 KB) [file 425_2024_4458_MOESM4_ESM.doc]

**Table S4. Tandem and segmental duplication genes of R2R3-MYB transcription factor gene family in *P. trichocarpa* genome**

| Gene name | Gene name | Duplication type |
| --- | --- | --- |
| PtrMYB039 | PtrMYB040 | Tandem |
| PtrMYB113 | PtrMYB114 | Tandem |
| PtrMYB114 | PtrMYB115 | Tandem |
| PtrMYB129 | PtrMYB130 | Tandem |
| PtrMYB135 | PtrMYB136 | Tandem |
| PtrMYB164 | PtrMYB165 | Tandem |
| PtrMYB165 | PtrMYB166 | Tandem |
| PtrMYB166 | PtrMYB167 | Tandem |
| PtrMYB167 | PtrMYB168 | Tandem |
| PtrMYB180 | PtrMYB181 | Tandem |
| PtrMYB187 | PtrMYB188 | Tandem |
| PtrMYB188 | PtrMYB189 | Tandem |
| PtrMYB001 | PtrMYB043 | WGD or Segmental |
| PtrMYB001 | PtrMYB129 | WGD or Segmental |
| PtrMYB001 | PtrMYB179 | WGD or Segmental |
| PtrMYB002 | PtrMYB042 | WGD or Segmental |
| PtrMYB003 | PtrMYB041 | WGD or Segmental |
| PtrMYB004 | PtrMYB029 | WGD or Segmental |
| PtrMYB004 | PtrMYB039 | WGD or Segmental |
| PtrMYB004 | PtrMYB141 | WGD or Segmental |
| PtrMYB005 | PtrMYB038 | WGD or Segmental |
| PtrMYB006 | PtrMYB037 | WGD or Segmental |
| PtrMYB006 | PtrMYB125 | WGD or Segmental |
| PtrMYB006 | PtrMYB152 | WGD or Segmental |
| PtrMYB007 | PtrMYB036 | WGD or Segmental |
| PtrMYB008 | PtrMYB034 | WGD or Segmental |
| PtrMYB010 | PtrMYB095 | WGD or Segmental |
| PtrMYB011 | PtrMYB096 | WGD or Segmental |
| PtrMYB012 | PtrMYB097 | WGD or Segmental |
| PtrMYB013 | PtrMYB098 | WGD or Segmental |
| PtrMYB014 | PtrMYB098 | WGD or Segmental |
| PtrMYB014 | PtrMYB099 | WGD or Segmental |
| PtrMYB015 | PtrMYB025 | WGD or Segmental |
| PtrMYB015 | PtrMYB076 | WGD or Segmental |
| PtrMYB015 | PtrMYB100 | WGD or Segmental |
| PtrMYB015 | PtrMYB137 | WGD or Segmental |
| PtrMYB018 | PtrMYB150 | WGD or Segmental |
| PtrMYB019 | PtrMYB045 | WGD or Segmental |
| PtrMYB019 | PtrMYB116 | WGD or Segmental |
| PtrMYB019 | PtrMYB117 | WGD or Segmental |
| PtrMYB019 | PtrMYB134 | WGD or Segmental |
| PtrMYB019 | PtrMYB186 | WGD or Segmental |
| PtrMYB020 | PtrMYB118 | WGD or Segmental |
| PtrMYB021 | PtrMYB064 | WGD or Segmental |
| PtrMYB021 | PtrMYB091 | WGD or Segmental |
| PtrMYB022 | PtrMYB056 | WGD or Segmental |
| PtrMYB022 | PtrMYB063 | WGD or Segmental |
| PtrMYB022 | PtrMYB079 | WGD or Segmental |
| PtrMYB023 | PtrMYB057 | WGD or Segmental |
| PtrMYB023 | PtrMYB062 | WGD or Segmental |
| PtrMYB023 | PtrMYB078 | WGD or Segmental |
| PtrMYB024 | PtrMYB075 | WGD or Segmental |
| PtrMYB025 | PtrMYB061 | WGD or Segmental |
| PtrMYB025 | PtrMYB076 | WGD or Segmental |
| PtrMYB025 | PtrMYB100 | WGD or Segmental |
| PtrMYB025 | PtrMYB137 | WGD or Segmental |
| PtrMYB026 | PtrMYB138 | WGD or Segmental |
| PtrMYB027 | PtrMYB139 | WGD or Segmental |
| PtrMYB028 | PtrMYB127 | WGD or Segmental |
| PtrMYB028 | PtrMYB140 | WGD or Segmental |
| PtrMYB028 | PtrMYB154 | WGD or Segmental |
| PtrMYB029 | PtrMYB039 | WGD or Segmental |
| PtrMYB029 | PtrMYB141 | WGD or Segmental |
| PtrMYB030 | PtrMYB055 | WGD or Segmental |
| PtrMYB030 | PtrMYB128 | WGD or Segmental |
| PtrMYB030 | PtrMYB142 | WGD or Segmental |
| PtrMYB031 | PtrMYB087 | WGD or Segmental |
| PtrMYB031 | PtrMYB108 | WGD or Segmental |
| PtrMYB031 | PtrMYB143 | WGD or Segmental |
| PtrMYB032 | PtrMYB107 | WGD or Segmental |
| PtrMYB032 | PtrMYB144 | WGD or Segmental |
| PtrMYB037 | PtrMYB125 | WGD or Segmental |
| PtrMYB037 | PtrMYB152 | WGD or Segmental |
| PtrMYB039 | PtrMYB141 | WGD or Segmental |
| PtrMYB043 | PtrMYB129 | WGD or Segmental |
| PtrMYB043 | PtrMYB179 | WGD or Segmental |
| PtrMYB044 | PtrMYB113 | WGD or Segmental |
| PtrMYB045 | PtrMYB116 | WGD or Segmental |
| PtrMYB045 | PtrMYB117 | WGD or Segmental |
| PtrMYB045 | PtrMYB134 | WGD or Segmental |
| PtrMYB046 | PtrMYB170 | WGD or Segmental |
| PtrMYB047 | PtrMYB169 | WGD or Segmental |
| PtrMYB048 | PtrMYB163 | WGD or Segmental |
| PtrMYB049 | PtrMYB162 | WGD or Segmental |
| PtrMYB050 | PtrMYB162 | WGD or Segmental |
| PtrMYB051 | PtrMYB159 | WGD or Segmental |
| PtrMYB051 | PtrMYB161 | WGD or Segmental |
| PtrMYB052 | PtrMYB190 | WGD or Segmental |
| PtrMYB052 | PtrMYB190.1 | WGD or Segmental |
| PtrMYB053 | PtrMYB101 | WGD or Segmental |
| PtrMYB054 | PtrMYB068 | WGD or Segmental |
| PtrMYB054 | PtrMYB094 | WGD or Segmental |
| PtrMYB055 | PtrMYB128 | WGD or Segmental |
| PtrMYB055 | PtrMYB142 | WGD or Segmental |
| PtrMYB056 | PtrMYB063 | WGD or Segmental |
| PtrMYB056 | PtrMYB079 | WGD or Segmental |
| PtrMYB057 | PtrMYB062 | WGD or Segmental |
| PtrMYB057 | PtrMYB078 | WGD or Segmental |
| PtrMYB058 | PtrMYB077 | WGD or Segmental |
| PtrMYB061 | PtrMYB076 | WGD or Segmental |
| PtrMYB061 | PtrMYB100 | WGD or Segmental |
| PtrMYB061 | PtrMYB137 | WGD or Segmental |
| PtrMYB062 | PtrMYB078 | WGD or Segmental |
| PtrMYB063 | PtrMYB079 | WGD or Segmental |
| PtrMYB064 | PtrMYB091 | WGD or Segmental |
| PtrMYB065 | PtrMYB177 | WGD or Segmental |
| PtrMYB068 | PtrMYB094 | WGD or Segmental |
| PtrMYB068 | PtrMYB155 | WGD or Segmental |
| PtrMYB069 | PtrMYB176 | WGD or Segmental |
| PtrMYB070 | PtrMYB172 | WGD or Segmental |
| PtrMYB071 | PtrMYB173 | WGD or Segmental |
| PtrMYB073 | PtrMYB175 | WGD or Segmental |
| PtrMYB074 | PtrMYB171 | WGD or Segmental |
| PtrMYB076 | PtrMYB100 | WGD or Segmental |
| PtrMYB076 | PtrMYB137 | WGD or Segmental |
| PtrMYB080 | PtrMYB156 | WGD or Segmental |
| PtrMYB081 | PtrMYB112 | WGD or Segmental |
| PtrMYB083 | PtrMYB111 | WGD or Segmental |
| PtrMYB084 | PtrMYB110 | WGD or Segmental |
| PtrMYB084 | PtrMYB160 | WGD or Segmental |
| PtrMYB085 | PtrMYB109 | WGD or Segmental |
| PtrMYB085 | PtrMYB161 | WGD or Segmental |
| PtrMYB087 | PtrMYB108 | WGD or Segmental |
| PtrMYB087 | PtrMYB143 | WGD or Segmental |
| PtrMYB088 | PtrMYB106 | WGD or Segmental |
| PtrMYB088 | PtrMYB120 | WGD or Segmental |
| PtrMYB089 | PtrMYB105 | WGD or Segmental |
| PtrMYB090 | PtrMYB104 | WGD or Segmental |
| PtrMYB092 | PtrMYB103 | WGD or Segmental |
| PtrMYB094 | PtrMYB155 | WGD or Segmental |
| PtrMYB100 | PtrMYB137 | WGD or Segmental |
| PtrMYB105 | PtrMYB169 | WGD or Segmental |
| PtrMYB106 | PtrMYB120 | WGD or Segmental |
| PtrMYB106 | PtrMYB147 | WGD or Segmental |
| PtrMYB107 | PtrMYB144 | WGD or Segmental |
| PtrMYB108 | PtrMYB143 | WGD or Segmental |
| PtrMYB109 | PtrMYB161 | WGD or Segmental |
| PtrMYB110 | PtrMYB160 | WGD or Segmental |
| PtrMYB116 | PtrMYB117 | WGD or Segmental |
| PtrMYB116 | PtrMYB134 | WGD or Segmental |
| PtrMYB116 | PtrMYB186 | WGD or Segmental |
| PtrMYB119 | PtrMYB145 | WGD or Segmental |
| PtrMYB120 | PtrMYB147 | WGD or Segmental |
| PtrMYB121 | PtrMYB148 | WGD or Segmental |
| PtrMYB121 | PtrMYB158 | WGD or Segmental |
| PtrMYB122 | PtrMYB149 | WGD or Segmental |
| PtrMYB123 | PtrMYB150 | WGD or Segmental |
| PtrMYB124 | PtrMYB151 | WGD or Segmental |
| PtrMYB125 | PtrMYB152 | WGD or Segmental |
| PtrMYB126 | PtrMYB153 | WGD or Segmental |
| PtrMYB127 | PtrMYB154 | WGD or Segmental |
| PtrMYB128 | PtrMYB142 | WGD or Segmental |
| PtrMYB129 | PtrMYB179 | WGD or Segmental |
| PtrMYB131 | PtrMYB182 | WGD or Segmental |
| PtrMYB132 | PtrMYB183 | WGD or Segmental |
| PtrMYB133 | PtrMYB185 | WGD or Segmental |
| PtrMYB134 | PtrMYB186 | WGD or Segmental |
| PtrMYB135 | PtrMYB187 | WGD or Segmental |
| PtrMYB140 | PtrMYB154 | WGD or Segmental |
| PtrMYB190 | PtrMYB190.1 | WGD or Segmental |
