# Supplementary material for: Genome-wide analysis of R2R3-MYB transcription factors in poplar and functional validation of PagMYB147 in defense against Melampsora magnusiana
Source: Planta. 2024 Jul 6;260(2):47. doi: 10.1007/s00425-024-04458-3 (PMC11227472; doi:10.1007/s00425-024-04458-3)
Supplement: Supplementary file 5 — Supplementary file5 (DOC 24 KB) [file 425_2024_4458_MOESM5_ESM.doc]

**Table S5. Syntenic relationships of R2R3-MYBs between *P. trichocarpa* and *O. sativa***

| *P. trichocarpa* | *O. sativa* |
| --- | --- |
| Gene name | Gene ID |
| PtrMYB008 | LOC_Os03g29614.1 |
| PtrMYB011 | LOC_Os01g74410.1 |
| PtrMYB011 | LOC_Os11g47460.1 |
| PtrMYB012 | LOC_Os01g63680.1 |
| PtrMYB015 | LOC_Os06g43090.1 |
| PtrMYB019 | LOC_Os07g37210.1 |
| PtrMYB021 | LOC_Os04g43680.1 |
| PtrMYB023 | LOC_Os02g51799.1 |
| PtrMYB023 | LOC_Os03g27090.1 |
| PtrMYB023 | LOC_Os06g11780.1 |
| PtrMYB023 | LOC_Os08g37970.1 |
| PtrMYB024 | LOC_Os03g56090.1 |
| PtrMYB027 | LOC_Os04g46384.1 |
| PtrMYB028 | LOC_Os02g42870.1 |
| PtrMYB028 | LOC_Os04g45060.1 |
| PtrMYB030 | LOC_Os01g18240.1 |
| PtrMYB030 | LOC_Os05g04820.1 |
| PtrMYB031 | LOC_Os01g19330.1 |
| PtrMYB031 | LOC_Os05g04210.1 |
| PtrMYB032 | LOC_Os01g19970.1 |
| PtrMYB032 | LOC_Os03g19120.1 |
| PtrMYB034 | LOC_Os03g29614.1 |
| PtrMYB045 | LOC_Os07g37210.1 |
| PtrMYB048 | LOC_Os01g03720.1 |
| PtrMYB053 | LOC_Os08g43550.1 |
| PtrMYB053 | LOC_Os09g36730.1 |
| PtrMYB055 | LOC_Os01g18240.1 |
| PtrMYB055 | LOC_Os05g04820.1 |
| PtrMYB057 | LOC_Os02g51799.1 |
| PtrMYB057 | LOC_Os03g27090.1 |
| PtrMYB057 | LOC_Os06g11780.1 |
| PtrMYB059 | LOC_Os09g36730.1 |
| PtrMYB062 | LOC_Os02g51799.1 |
| PtrMYB062 | LOC_Os03g27090.1 |
| PtrMYB062 | LOC_Os06g11780.1 |
| PtrMYB062 | LOC_Os08g37970.1 |
| PtrMYB064 | LOC_Os04g43680.1 |
| PtrMYB069 | LOC_Os02g54520.1 |
| PtrMYB069 | LOC_Os08g15020.1 |
| PtrMYB072 | LOC_Os06g10350.1 |
| PtrMYB075 | LOC_Os03g56090.1 |
| PtrMYB076 | LOC_Os09g01960.1 |
| PtrMYB078 | LOC_Os02g51799.1 |
| PtrMYB078 | LOC_Os03g27090.1 |
| PtrMYB078 | LOC_Os06g11780.1 |
| PtrMYB078 | LOC_Os08g37970.1 |
| PtrMYB081 | LOC_Os01g63160.1 |
| PtrMYB084 | LOC_Os08g33800.1 |
| PtrMYB085 | LOC_Os02g36890.1 |
| PtrMYB085 | LOC_Os08g33660.1 |
| PtrMYB087 | LOC_Os01g19330.1 |
| PtrMYB087 | LOC_Os03g20090.1 |
| PtrMYB087 | LOC_Os12g37690.1 |
| PtrMYB091 | LOC_Os02g41510.1 |
| PtrMYB091 | LOC_Os04g43680.1 |
| PtrMYB091 | LOC_Os10g33810.1 |
| PtrMYB092 | LOC_Os03g04900.1 |
| PtrMYB095 | LOC_Os05g41166.1 |
| PtrMYB096 | LOC_Os01g74410.1 |
| PtrMYB096 | LOC_Os11g47460.1 |
| PtrMYB096 | LOC_Os12g37970.1 |
| PtrMYB097 | LOC_Os01g63680.1 |
| PtrMYB100 | LOC_Os06g43090.1 |
| PtrMYB100 | LOC_Os09g01960.1 |
| PtrMYB101 | LOC_Os08g43550.1 |
| PtrMYB101 | LOC_Os09g36730.1 |
| PtrMYB102 | LOC_Os01g18240.1 |
| PtrMYB102 | LOC_Os05g04820.1 |
| PtrMYB102 | LOC_Os07g44090.3 |
| PtrMYB103 | LOC_Os03g04900.1 |
| PtrMYB106 | LOC_Os01g03720.1 |
| PtrMYB107 | LOC_Os03g19120.1 |
| PtrMYB108 | LOC_Os03g20090.1 |
| PtrMYB108 | LOC_Os12g37690.1 |
| PtrMYB109 | LOC_Os02g36890.1 |
| PtrMYB109 | LOC_Os08g33660.1 |
| PtrMYB112 | LOC_Os01g63160.1 |
| PtrMYB116 | LOC_Os07g37210.1 |
| PtrMYB117 | LOC_Os07g37210.1 |
| PtrMYB119 | LOC_Os03g51110.1 |
| PtrMYB121 | LOC_Os04g38740.1 |
| PtrMYB124 | LOC_Os01g07450.1 |
| PtrMYB126 | LOC_Os02g42850.2 |
| PtrMYB127 | LOC_Os02g42870.1 |
| PtrMYB128 | LOC_Os01g18240.1 |
| PtrMYB128 | LOC_Os05g04820.1 |
| PtrMYB128 | LOC_Os07g44090.3 |
| PtrMYB134 | LOC_Os07g37210.1 |
| PtrMYB139 | LOC_Os04g46384.1 |
| PtrMYB140 | LOC_Os02g42870.1 |
| PtrMYB140 | LOC_Os04g45060.1 |
| PtrMYB142 | LOC_Os01g18240.1 |
| PtrMYB142 | LOC_Os05g04820.1 |
| PtrMYB143 | LOC_Os01g19330.1 |
| PtrMYB143 | LOC_Os05g04210.1 |
| PtrMYB144 | LOC_Os01g19970.1 |
| PtrMYB144 | LOC_Os03g19120.1 |
| PtrMYB151 | LOC_Os01g07450.1 |
| PtrMYB151 | LOC_Os01g36460.1 |
| PtrMYB153 | LOC_Os04g45020.1 |
| PtrMYB159 | LOC_Os03g26130.1 |
| PtrMYB161 | LOC_Os04g38740.1 |
| PtrMYB163 | LOC_Os01g03720.1 |
| PtrMYB176 | LOC_Os02g54520.1 |
| PtrMYB176 | LOC_Os08g15020.1 |
| PtrMYB176 | LOC_Os09g26170.1 |
| PtrMYB178 | LOC_Os10g33810.1 |
| PtrMYB186 | LOC_Os07g37210.1 |
| PtrMYB008 | LOC_Os03g29614.1 |
| PtrMYB011 | LOC_Os01g74410.1 |
| PtrMYB011 | LOC_Os11g47460.1 |
| PtrMYB012 | LOC_Os01g63680.1 |
| PtrMYB015 | LOC_Os06g43090.1 |
| PtrMYB019 | LOC_Os07g37210.1 |
| PtrMYB021 | LOC_Os04g43680.1 |
| PtrMYB023 | LOC_Os02g51799.1 |
| PtrMYB023 | LOC_Os03g27090.1 |
| PtrMYB023 | LOC_Os06g11780.1 |
| PtrMYB023 | LOC_Os08g37970.1 |
| PtrMYB024 | LOC_Os03g56090.1 |
| PtrMYB027 | LOC_Os04g46384.1 |
| PtrMYB028 | LOC_Os02g42870.1 |
| PtrMYB028 | LOC_Os04g45060.1 |
| PtrMYB030 | LOC_Os01g18240.1 |
| PtrMYB030 | LOC_Os05g04820.1 |
| PtrMYB031 | LOC_Os01g19330.1 |
| PtrMYB031 | LOC_Os05g04210.1 |
| PtrMYB032 | LOC_Os01g19970.1 |
| PtrMYB032 | LOC_Os03g19120.1 |
| PtrMYB034 | LOC_Os03g29614.1 |
| PtrMYB045 | LOC_Os07g37210.1 |
| PtrMYB048 | LOC_Os01g03720.1 |
| PtrMYB053 | LOC_Os08g43550.1 |
| PtrMYB053 | LOC_Os09g36730.1 |
| PtrMYB055 | LOC_Os01g18240.1 |
| PtrMYB055 | LOC_Os05g04820.1 |
| PtrMYB057 | LOC_Os02g51799.1 |
| PtrMYB057 | LOC_Os03g27090.1 |
| PtrMYB057 | LOC_Os06g11780.1 |
| PtrMYB059 | LOC_Os09g36730.1 |
| PtrMYB062 | LOC_Os02g51799.1 |
| PtrMYB062 | LOC_Os03g27090.1 |
| PtrMYB062 | LOC_Os06g11780.1 |
| PtrMYB062 | LOC_Os08g37970.1 |
| PtrMYB064 | LOC_Os04g43680.1 |
| PtrMYB069 | LOC_Os02g54520.1 |
| PtrMYB069 | LOC_Os08g15020.1 |
| PtrMYB072 | LOC_Os06g10350.1 |
| PtrMYB075 | LOC_Os03g56090.1 |
| PtrMYB076 | LOC_Os09g01960.1 |
| PtrMYB078 | LOC_Os02g51799.1 |
| PtrMYB078 | LOC_Os03g27090.1 |
| PtrMYB078 | LOC_Os06g11780.1 |
| PtrMYB078 | LOC_Os08g37970.1 |
| PtrMYB081 | LOC_Os01g63160.1 |
| PtrMYB084 | LOC_Os08g33800.1 |
| PtrMYB085 | LOC_Os02g36890.1 |
| PtrMYB085 | LOC_Os08g33660.1 |
| PtrMYB087 | LOC_Os01g19330.1 |
| PtrMYB087 | LOC_Os03g20090.1 |
| PtrMYB087 | LOC_Os12g37690.1 |
| PtrMYB091 | LOC_Os02g41510.1 |
| PtrMYB091 | LOC_Os04g43680.1 |
| PtrMYB091 | LOC_Os10g33810.1 |
| PtrMYB092 | LOC_Os03g04900.1 |
| PtrMYB095 | LOC_Os05g41166.1 |
| PtrMYB096 | LOC_Os01g74410.1 |
| PtrMYB096 | LOC_Os11g47460.1 |
| PtrMYB096 | LOC_Os12g37970.1 |
| PtrMYB097 | LOC_Os01g63680.1 |
| PtrMYB100 | LOC_Os06g43090.1 |
| PtrMYB100 | LOC_Os09g01960.1 |
| PtrMYB101 | LOC_Os08g43550.1 |
| PtrMYB101 | LOC_Os09g36730.1 |
| PtrMYB102 | LOC_Os01g18240.1 |
| PtrMYB102 | LOC_Os05g04820.1 |
| PtrMYB102 | LOC_Os07g44090.3 |
| PtrMYB103 | LOC_Os03g04900.1 |
| PtrMYB106 | LOC_Os01g03720.1 |
| PtrMYB107 | LOC_Os03g19120.1 |
| PtrMYB108 | LOC_Os03g20090.1 |
| PtrMYB108 | LOC_Os12g37690.1 |
| PtrMYB109 | LOC_Os02g36890.1 |
| PtrMYB109 | LOC_Os08g33660.1 |
| PtrMYB112 | LOC_Os01g63160.1 |
| PtrMYB116 | LOC_Os07g37210.1 |
| PtrMYB117 | LOC_Os07g37210.1 |
| PtrMYB119 | LOC_Os03g51110.1 |
| PtrMYB121 | LOC_Os04g38740.1 |
| PtrMYB124 | LOC_Os01g07450.1 |
| PtrMYB126 | LOC_Os02g42850.2 |
| PtrMYB127 | LOC_Os02g42870.1 |
| PtrMYB128 | LOC_Os01g18240.1 |
| PtrMYB128 | LOC_Os05g04820.1 |
| PtrMYB128 | LOC_Os07g44090.3 |
| PtrMYB134 | LOC_Os07g37210.1 |
| PtrMYB139 | LOC_Os04g46384.1 |
| PtrMYB140 | LOC_Os02g42870.1 |
| PtrMYB140 | LOC_Os04g45060.1 |
| PtrMYB142 | LOC_Os01g18240.1 |
| PtrMYB142 | LOC_Os05g04820.1 |
| PtrMYB143 | LOC_Os01g19330.1 |
| PtrMYB143 | LOC_Os05g04210.1 |
| PtrMYB144 | LOC_Os01g19970.1 |
| PtrMYB144 | LOC_Os03g19120.1 |
| PtrMYB151 | LOC_Os01g07450.1 |
| PtrMYB151 | LOC_Os01g36460.1 |
| PtrMYB153 | LOC_Os04g45020.1 |
| PtrMYB159 | LOC_Os03g26130.1 |
| PtrMYB161 | LOC_Os04g38740.1 |
| PtrMYB163 | LOC_Os01g03720.1 |
| PtrMYB176 | LOC_Os02g54520.1 |
| PtrMYB176 | LOC_Os08g15020.1 |
| PtrMYB176 | LOC_Os09g26170.1 |
| PtrMYB178 | LOC_Os10g33810.1 |
| PtrMYB186 | LOC_Os07g37210.1 |
