# Supplementary material for: Genome-wide analysis of R2R3-MYB transcription factors in poplar and functional validation of PagMYB147 in defense against Melampsora magnusiana
Source: Planta. 2024 Jul 6;260(2):47. doi: 10.1007/s00425-024-04458-3 (PMC11227472; doi:10.1007/s00425-024-04458-3)
Supplement: Supplementary file 6 — Supplementary file6 (DOC 26 KB) [file 425_2024_4458_MOESM6_ESM.doc]

**Table S6. Syntenic relationships of R2R3-MYBs**

**between *P. trichocarpa* and *A. thaliana***

| *P. trichocarpa* | *A. thaliana* | |
| --- | --- | --- |
| Gene name | Gene ID | Gene name |
| PtrMYB001 | AT3G13540.1 | AtMYB005 |
| PtrMYB005 | AT1G63910.1 | AtMYB103 |
| PtrMYB006 | AT4G12350.1 | AtMYB042 |
| PtrMYB006 | AT4G22680.1 | AtMYB085 |
| PtrMYB006 | AT5G62320.1 | AtMYB099 |
| PtrMYB007 | AT4G17785.1 | AtMYB039 |
| PtrMYB007 | AT5G16770.2 | AtMYB009 |
| PtrMYB009 | AT3G13890.1 | AtMYB026 |
| PtrMYB010 | AT2G26950.1 | AtMYB104 |
| PtrMYB010 | AT2G26960.1 | AtMYB081 |
| PtrMYB011 | AT3G46130.1 | AtMYB048 |
| PtrMYB011 | AT5G59780.3 | AtMYB059 |
| PtrMYB012 | AT5G58850.1 | AtMYB119 |
| PtrMYB013 | AT5G12870.1 | AtMYB046 |
| PtrMYB014 | AT3G08500.1 | AtMYB083 |
| PtrMYB015 | AT2G23290.1 | AtMYB070 |
| PtrMYB016 | AT5G14340.1 | AtMYB040 |
| PtrMYB017 | AT3G27810.1 | AtMYB021 |
| PtrMYB018 | AT3G27785.1 | AtMYB118 |
| PtrMYB018 | AT5G40430.1 | AtMYB022 |
| PtrMYB019 | AT4G21440.1 | AtMYB102 |
| PtrMYB019 | AT5G54230.1 | AtMYB049 |
| PtrMYB020 | AT5G56110.1 | AtMYB080 |
| PtrMYB021 | AT2G31180.1 | AtMYB014 |
| PtrMYB021 | AT3G23250.1 | AtMYB015 |
| PtrMYB023 | AT1G34670.1 | AtMYB093 |
| PtrMYB023 | AT5G10280.1 | AtMYB092 |
| PtrMYB023 | AT5G65230.1 | AtMYB053 |
| PtrMYB024 | AT3G49690.1 | AtMYB084 |
| PtrMYB024 | AT4G37780.1 | AtMYB087 |
| PtrMYB025 | AT2G23290.1 | AtMYB070 |
| PtrMYB025 | AT3G50060.1 | AtMYB077 |
| PtrMYB025 | AT4G37260.1 | AtMYB073 |
| PtrMYB025 | AT5G67300.1 | AtMYB044 |
| PtrMYB026 | AT4G37260.1 | AtMYB073 |
| PtrMYB027 | AT3G60460.1 | AtMYB125 |
| PtrMYB028 | AT3G61250.1 | AtMYB017 |
| PtrMYB030 | AT4G01680.2 | AtMYB055 |
| PtrMYB031 | AT2G47190.1 | AtMYB002 |
| PtrMYB031 | AT3G06490.1 | AtMYB108 |
| PtrMYB032 | AT2G47460.1 | AtMYB012 |
| PtrMYB032 | AT3G62610.1 | AtMYB011 |
| PtrMYB032 | AT5G49330.1 | AtMYB111 |
| PtrMYB033 | AT2G32460.1 | AtMYB101 |
| PtrMYB033 | AT5G55020.1 | AtMYB120 |
| PtrMYB036 | AT4G17785.1 | AtMYB039 |
| PtrMYB036 | AT5G16770.2 | AtMYB009 |
| PtrMYB037 | AT4G12350.1 | AtMYB042 |
| PtrMYB037 | AT4G22680.1 | AtMYB085 |
| PtrMYB037 | AT5G62320.1 | AtMYB099 |
| PtrMYB038 | AT1G63910.1 | AtMYB103 |
| PtrMYB043 | AT3G13540.1 | AtMYB005 |
| PtrMYB044 | AT4G21440.1 | AtMYB102 |
| PtrMYB045 | AT4G05100.1 | AtMYB074 |
| PtrMYB045 | AT4G21440.1 | AtMYB102 |
| PtrMYB046 | AT1G66230.1 | AtMYB020 |
| PtrMYB046 | AT5G16600.1 | AtMYB043 |
| PtrMYB049 | AT3G30210.1 | AtMYB121 |
| PtrMYB050 | AT3G30210.1 | AtMYB121 |
| PtrMYB051 | AT3G28910.1 | AtMYB030 |
| PtrMYB053 | AT2G16720.1 | AtMYB007 |
| PtrMYB053 | AT4G34990.1 | AtMYB032 |
| PtrMYB053 | AT4G38620.1 | AtMYB004 |
| PtrMYB054 | AT5G23000.1 | AtMYB037 |
| PtrMYB055 | AT1G09540.1 | AtMYB061 |
| PtrMYB055 | AT1G57560.1 | AtMYB050 |
| PtrMYB055 | AT5G26660.1 | AtMYB086 |
| PtrMYB057 | AT5G10280.1 | AtMYB092 |
| PtrMYB057 | AT5G65230.1 | AtMYB053 |
| PtrMYB058 | AT1G16490.1 | AtMYB058 |
| PtrMYB058 | AT1G79180.2 | AtMYB063 |
| PtrMYB058 | AT3G12820.1 | AtMYB010 |
| PtrMYB059 | AT4G34990.1 | AtMYB032 |
| PtrMYB059 | AT4G38620.1 | AtMYB004 |
| PtrMYB061 | AT2G23290.1 | AtMYB070 |
| PtrMYB061 | AT3G50060.1 | AtMYB077 |
| PtrMYB061 | AT4G37260.1 | AtMYB073 |
| PtrMYB061 | AT5G67300.1 | AtMYB044 |
| PtrMYB062 | AT1G34670.1 | AtMYB093 |
| PtrMYB062 | AT5G10280.1 | AtMYB092 |
| PtrMYB062 | AT5G65230.1 | AtMYB053 |
| PtrMYB064 | AT2G31180.1 | AtMYB014 |
| PtrMYB064 | AT3G23250.1 | AtMYB015 |
| PtrMYB066 | AT2G37630.1 | AtMYB091 |
| PtrMYB067 | AT3G53200.1 | AtMYB027 |
| PtrMYB068 | AT2G36890.2 | AtMYB038 |
| PtrMYB069 | AT5G57620.1 | AtMYB036 |
| PtrMYB075 | AT3G49690.1 | AtMYB084 |
| PtrMYB075 | AT4G37780.1 | AtMYB087 |
| PtrMYB075 | AT5G65790.1 | AtMYB068 |
| PtrMYB076 | AT2G23290.1 | AtMYB070 |
| PtrMYB076 | AT3G50060.1 | AtMYB077 |
| PtrMYB076 | AT4G37260.1 | AtMYB073 |
| PtrMYB076 | AT5G67300.1 | AtMYB044 |
| PtrMYB077 | AT1G16490.1 | AtMYB058 |
| PtrMYB077 | AT1G79180.2 | AtMYB063 |
| PtrMYB078 | AT5G10280.1 | AtMYB092 |
| PtrMYB078 | AT5G65230.1 | AtMYB053 |
| PtrMYB081 | AT2G39880.1 | AtMYB025 |
| PtrMYB081 | AT3G55730.1 | AtMYB109 |
| PtrMYB083 | AT3G12720.1 | AtMYB067 |
| PtrMYB084 | AT1G26780.2 | AtMYB117 |
| PtrMYB084 | AT1G69560.2 | AtMYB105 |
| PtrMYB084 | AT3G29020.2 | AtMYB110 |
| PtrMYB087 | AT1G48000.1 | AtMYB112 |
| PtrMYB087 | AT2G47190.1 | AtMYB002 |
| PtrMYB087 | AT3G06490.1 | AtMYB108 |
| PtrMYB087 | AT5G49620.2 | AtMYB078 |
| PtrMYB088 | AT1G68320.1 | AtMYB062 |
| PtrMYB091 | AT3G23250.1 | AtMYB015 |
| PtrMYB092 | AT4G13480.1 | AtMYB079 |
| PtrMYB094 | AT2G36890.2 | AtMYB038 |
| PtrMYB094 | AT5G23000.1 | AtMYB037 |
| PtrMYB096 | AT3G46130.1 | AtMYB048 |
| PtrMYB096 | AT5G59780.3 | AtMYB059 |
| PtrMYB097 | AT5G58850.1 | AtMYB119 |
| PtrMYB098 | AT5G12870.1 | AtMYB046 |
| PtrMYB099 | AT3G08500.1 | AtMYB083 |
| PtrMYB101 | AT2G16720.1 | AtMYB007 |
| PtrMYB101 | AT4G34990.1 | AtMYB032 |
| PtrMYB101 | AT4G38620.1 | AtMYB004 |
| PtrMYB103 | AT4G13480.1 | AtMYB079 |
| PtrMYB106 | AT1G68320.1 | AtMYB062 |
| PtrMYB107 | AT5G49330.1 | AtMYB111 |
| PtrMYB108 | AT1G48000.1 | AtMYB112 |
| PtrMYB108 | AT3G06490.1 | AtMYB108 |
| PtrMYB108 | AT5G49620.2 | AtMYB078 |
| PtrMYB109 | AT5G61420.2 | AtMYB028 |
| PtrMYB110 | AT1G26780.2 | AtMYB117 |
| PtrMYB110 | AT1G69560.2 | AtMYB105 |
| PtrMYB110 | AT3G29020.2 | AtMYB110 |
| PtrMYB111 | AT3G12720.1 | AtMYB067 |
| PtrMYB112 | AT2G39880.1 | AtMYB025 |
| PtrMYB112 | AT3G55730.1 | AtMYB109 |
| PtrMYB113 | AT4G21440.1 | AtMYB102 |
| PtrMYB116 | AT4G05100.1 | AtMYB074 |
| PtrMYB116 | AT4G21440.1 | AtMYB102 |
| PtrMYB117 | AT5G54230.1 | AtMYB049 |
| PtrMYB118 | AT5G56110.1 | AtMYB080 |
| PtrMYB119 | AT1G73410.1 | AtMYB054 |
| PtrMYB122 | AT3G27920.1 | AtMYB000 |
| PtrMYB122 | AT5G40330.1 | AtMYB023 |
| PtrMYB123 | AT3G27785.1 | AtMYB118 |
| PtrMYB123 | AT5G40360.1 | AtMYB115 |
| PtrMYB125 | AT4G12350.1 | AtMYB042 |
| PtrMYB125 | AT4G22680.1 | AtMYB085 |
| PtrMYB125 | AT5G62320.1 | AtMYB099 |
| PtrMYB126 | AT3G48920.1 | AtMYB045 |
| PtrMYB126 | AT4G25560.1 | AtMYB018 |
| PtrMYB126 | AT5G52260.1 | AtMYB019 |
| PtrMYB128 | AT1G09540.1 | AtMYB061 |
| PtrMYB128 | AT1G57560.1 | AtMYB050 |
| PtrMYB128 | AT5G26660.1 | AtMYB086 |
| PtrMYB131 | AT5G17800.1 | AtMYB056 |
| PtrMYB133 | AT1G22640.1 | AtMYB003 |
| PtrMYB133 | AT1G35515.1 | AtMYB008 |
| PtrMYB133 | AT4G09460.1 | AtMYB006 |
| PtrMYB134 | AT5G54230.1 | AtMYB049 |
| PtrMYB137 | AT2G23290.1 | AtMYB070 |
| PtrMYB137 | AT3G50060.1 | AtMYB077 |
| PtrMYB137 | AT5G67300.1 | AtMYB044 |
| PtrMYB140 | AT3G61250.1 | AtMYB017 |
| PtrMYB142 | AT4G01680.2 | AtMYB055 |
| PtrMYB143 | AT2G47190.1 | AtMYB002 |
| PtrMYB143 | AT5G49620.2 | AtMYB078 |
| PtrMYB144 | AT2G47460.1 | AtMYB012 |
| PtrMYB144 | AT3G62610.1 | AtMYB011 |
| PtrMYB144 | AT5G49330.1 | AtMYB111 |
| PtrMYB145 | AT1G73410.1 | AtMYB054 |
| PtrMYB149 | AT3G27920.1 | AtMYB000 |
| PtrMYB149 | AT5G40330.1 | AtMYB023 |
| PtrMYB150 | AT3G27785.1 | AtMYB118 |
| PtrMYB150 | AT5G40360.1 | AtMYB115 |
| PtrMYB152 | AT5G62320.1 | AtMYB099 |
| PtrMYB153 | AT3G48920.1 | AtMYB045 |
| PtrMYB153 | AT4G25560.1 | AtMYB018 |
| PtrMYB153 | AT5G52260.1 | AtMYB019 |
| PtrMYB155 | AT2G36890.2 | AtMYB038 |
| PtrMYB157 | AT3G01530.1 | AtMYB057 |
| PtrMYB157 | AT3G27810.1 | AtMYB021 |
| PtrMYB157 | AT5G40350.1 | AtMYB024 |
| PtrMYB158 | AT3G28470.1 | AtMYB035 |
| PtrMYB159 | AT3G28910.1 | AtMYB030 |
| PtrMYB160 | AT1G26780.2 | AtMYB117 |
| PtrMYB160 | AT1G69560.2 | AtMYB105 |
| PtrMYB160 | AT3G29020.2 | AtMYB110 |
| PtrMYB161 | AT3G01140.1 | AtMYB106 |
| PtrMYB161 | AT5G15310.1 | AtMYB016 |
| PtrMYB162 | AT3G30210.1 | AtMYB121 |
| PtrMYB164 | AT1G66370.1 | AtMYB113 |
| PtrMYB170 | AT1G66230.1 | AtMYB020 |
| PtrMYB176 | AT5G57620.1 | AtMYB036 |
| PtrMYB182 | AT5G17800.1 | AtMYB056 |
| PtrMYB184 | AT3G02940.1 | AtMYB107 |
| PtrMYB184 | AT5G16770.2 | AtMYB009 |
| PtrMYB185 | AT1G22640.1 | AtMYB003 |
| PtrMYB185 | AT1G35515.1 | AtMYB008 |
| PtrMYB185 | AT4G09460.1 | AtMYB006 |
