# Supplementary material for: Genome-wide analysis of R2R3-MYB transcription factors in poplar and functional validation of PagMYB147 in defense against Melampsora magnusiana
Source: Planta. 2024 Jul 6;260(2):47. doi: 10.1007/s00425-024-04458-3 (PMC11227472; doi:10.1007/s00425-024-04458-3)
Supplement: Supplementary file 7 — Supplementary file7 (DOC 17 KB) [file 425_2024_4458_MOESM7_ESM.doc]

**Table S7. Summary of motifs in R2R3-MYB transcription factors by MEME analysis**

| Name | Length | TF number | Sequence |
| --- | --- | --- | --- |
| Motif 1 | 50 | 189 | RCGKSCRLRWINYLRPD[IL]KRGN[FI][ST][PE][ED]E[ED][ED]LI[IL]RLH[AS]LLGN[RK]W[SA]LIA[AG]RLP |
| Motif 2 | 15 | 188 | GRTDNE[IV]KN[YH]WN[TS]H[LI] |
| Motif 3 | 21 | 191 | [KR][KR]GPW[TS]PEEDE[KL]LIXY[IV]XK[HY]G |
| Motif 4 | 11 | 153 | [NCR]WR[SA][LV]PKKAGL |
| Motif 5 | 21 | 63 | [KR]K[KR]L[IL][KS][MQR]GIDPVTH[KR]PLSDLX |
| Motif 6 | 11 | 92 | MGR[AS]PCC[DE]KVG |
| Motif 7 | 50 | 10 | [QT]K[QEHR][AI][KRT][QHN][LF][KN][CIL][DE][AVE][NQ][SN][KTY][QAL]F[KLRV][DQ][ATI][MIQV][RP][YES][FL][WM][MI][PS][RN][LI][IVL][QEK][KR][IA][GQES][ADGQ][ASE][SA][AEITY][SAI][SKPT][SV][ADGS][GMQT][TPS][SL][DIQ]S[PA][AEPS][DTV][SAY] |
| Motif 8 | 50 | 5 | [SF]P[LV][DE][EA]N[GA][RG]V[YD]R[TA][VT]ATR[VE][SIN]E[NK][FL]T[VIM]T[ND]L[DY]Q[SF]PNQV[AI][AD][DG][AG][TA][EK]NWP[NS][RC]DGF[NT][MK]G |
| Motif 9 | 30 | 8 | [AS][AP][SA][LT][SR]HM[AV]QWESAR[LV]EAEARL[SV]R[EQ]S[KLS]L[FIT][KLV]P |
| Motif 10 | 11 | 33 | P[RQ]NW[NS]LI[AS]EK[LI] |
