# Supplementary material for: Genome-wide analysis of R2R3-MYB transcription factors in poplar and functional validation of PagMYB147 in defense against Melampsora magnusiana
Source: Planta. 2024 Jul 6;260(2):47. doi: 10.1007/s00425-024-04458-3 (PMC11227472; doi:10.1007/s00425-024-04458-3)
Supplement: Supplementary file 8 — Supplementary file8 (DOC 14 KB) [file 425_2024_4458_MOESM8_ESM.doc]

**Table S8. The number of MBSs, MREs and MYB**

**in the 5’ regulatory sequences of poplar defense-related genes**

| Elements | Defense-related genes | | | | |
| --- | --- | --- | --- | --- | --- |
|  | *PtrCAT1* | *PtrSOD1* | *PtrPAL1* | *PtrPR5* | *PtrDefensin* |
| MBS (CAACTG) | - | 2 | - | 2 | - |
| MRE (AACCTAA) | 1 | - | - | 1 | 2 |
| MYB (TAACCA) | 4 | 5 | 2 | 8 | 2 |
